# Metabolic and Physiological Changes in the Roots of Two Oat Cultivars in Response to Complex Saline-Alkali Stress

**DOI:** 10.3389/fpls.2022.835414

**Published:** 2022-03-29

**Authors:** Yugang Gao, Yongling Jin, Wei Guo, Yingwen Xue, Lihe Yu

**Affiliations:** Heilongjiang Bayi Agricultural University, Daqing, China

**Keywords:** growth, metabolites, oat, saline-alkali stress, root

## Abstract

Saline-alkali stress is a major abiotic stress factor in agricultural productivity. Oat (*Avena sativa* L.) is a saline-alkali tolerant crop species. However, molecular mechanisms of saline-alkali tolerance in oats remain unclear. To understand the physiological and molecular mechanisms underlying seedling saline-alkali tolerance in oats, the phenotypic and metabolic responses of two oat cultivars, Baiyan7 (BY, tolerant cultivar) and Yizhangyan4 (YZY, sensitive cultivar), were characterized under saline-alkali stress conditions. Compared with YZY, BY showed better adaptability to saline-alkali stress. A total of 151 and 96 differential metabolites induced by saline-alkali stress were identified in roots of BY and YZY, respectively. More detailed analyses indicated that enhancements of energy metabolism and accumulations of organic acids were the active strategies of oat roots, in response to complex saline-alkali stress. The BY utilized sugars *via* sugar consumption more effectively, while amino acids strengthened metabolism and upregulated lignin and might be the positive responses of BY roots to saline-alkali stress, which led to a higher osmotic adjustment of solute concentrations and cell growth. The YZY mainly used soluble sugars and flavonoids combined with sugars to form glycosides, as osmotic regulatory substances or antioxidant substances, to cope with saline-alkali stress. The analyses of different metabolites of roots of tolerant and sensitive cultivars provided an important theoretical basis for understanding the mechanisms of saline-alkali tolerance and increased our knowledge of plant metabolism regulation under stress. Meanwhile, some related metabolites, such as proline, betaine, and *p*-coumaryl alcohol, can also be used as candidates for screening saline-alkali tolerant oat cultivars.

## Introduction

Oats (*Avena sativa* L.) is an important economic crop used for multiple purposes, such as in food, forage, and medicine. Because of its wide range of ecological adaptability, oats have become an irreplaceable grain and feed crop in ecologically fragile areas (Łabanowska et al., 2016), and have been widely used as an alternative crop for saline-alkali soil amelioration (Fu et al., 2011; Han et al., 2012).

Soil saline-alkalization is a worldwide environmental and ecological problem. The total area of saline-alkali soils in the world exceeds 10 × 10^8^ ha, and 10% of the total arable land is being affected by salinity (Shahid et al., 2018). In northeastern China, the Songnen Plain accounts for 3.73 × 10^6^ ha (accounting for 21% of the total area) and is one of the three typical soda saline-alkali soil distribution areas; in this area, soil salinization is mainly caused by sodium-carbonate (i.e., Na_2_CO_3_ and NaHCO_3_), which leads to increases in the pH of the soil to pH 10 in most areas (Yin et al., 2017; Fang et al., 2021). In addition, global warming, improper soil water-crop management, and pollution have led to constantly expanding areas undergoing salinization. According to the Food and Agricultural Organization, a manifested salinity is projected to affect 50% of global agricultural land by 2050 (Butcher et al., 2016). As two coexisting abiotic stresses, salt and alkali stress have severely restricted the development of global agriculture and food security (Bartels and Sunkar, 2005; Fang et al., 2021). Therefore, saline-alkalization is a major limiting factor for sustainable agricultural development and food security in the world. To solve this dilemma, developing and planting crop cultivars with high saline-alkali tolerance may be the most efficient approach.

The stress effects of soil saline-alkalization on plants include the effects of both salt stress and alkali stress. Salt stress, which results mainly from NaCl, Na_2_SO_4_, and other neutral salts, induces osmotic stress and ion injury by disrupting ion homeostasis and ion balance in plant cells (Fang et al., 2021; Wang et al., 2021). In addition, the superposition of osmotic stress and ionic stress can lead to secondary oxidative stress (Munns and Tester, 2008). The damage to plants caused by mixed salt-alkali stress is more serious (Rao et al., 2013), which is due to further increases in the pH during salt stress; a high pH, therefore, severely disturbs cell pH stability and destroys cell membrane integrity (Fang et al., 2021). It is well known that soil salinization and alkalization usually coexist. However, most studies have focused mainly on single salt stress (salt tress or alkali stress), and little attention has been given to combined saline-alkali stress.

Saline-alkali stress alters the physiological, biochemical, and molecular processes of plants (El-Esawi et al., 2019). Metabolomics studies can reflect the biological and physiological processes in response to stressful conditions at the cellular and molecular levels by monitoring the changes in metabolite levels and fluxes (Tinte et al., 2021). Therefore, metabolomics has become essential in studying the changes of metabolic pathways and tolerance mechanisms under environmental stress (Shao et al., 2011). Recent studies have shown that salinity induces several physiological processes, such as the osmotic and antioxidant adjustment defense mechanisms. To cope with osmotic stress, plants synthesize and accumulate compatible osmolytes, including organic acids, proline, betaine, *N*-containing compounds, sugars, straight-chain polyhydric polyols, and cyclic polyhydric alcohols (Yang et al., 2007; Zhao et al., 2013; Liu et al., 2015; Shen et al., 2018; Sun et al., 2019), to maintain cell turgor and osmotic potential. In oats (Liu et al., 2016), the salinity tolerance was associated with higher content of proline and soluble proteins. To cope with oxidative stress, the plant has two enzymatic and non-enzymatic active oxygen scavenging systems to avoid injury, which metabolizes reactive oxygen species (ROS) and their reaction products to avert oxidative stress conditions (Gill and Tuteja, 2010). Antioxidant enzymes mainly include superoxide dismutase (SOD), catalase (CAT), guaiacol peroxidase (GP), ascorbate peroxidase (APX), and glutathione reductase (GR) (Liu et al., 2020). Non-enzymatic antioxidants such as ascorbic acid, cysteine, carotenoids, alkaloids, α-tocopherol, flavonoids, and reduced glutathione can effectively help resist the damage caused by stress (Luo et al., 2009). Organic acids play an important role in maintaining the cell pH and ion balance (Fang et al., 2021). To cope with high pH, the accumulations of organic acids such as citrate, formate, lactate, acetate, succinate, malate, and oxalate, were observed in tomatoes (Wang et al., 2011), in *Chloris virgata* (Yang et al., 2010), and in grapevines (Xiang et al., 2019). Organic acids are synthesized to compensate for the deficiency of inorganic anions during saline-alkali stress, especially under alkaline stress (Guo et al., 2018), and they also play a role in osmotic regulation (Wang et al., 2021).

Oat crops are considered to be moderately tolerant to salinity and alkalinity, and some oat cultivars can grow in soil with pH values as high as 9 (Bai et al., 2018). However, the growth and physiological activities of oat plants are also affected by saline-alkali stress like other gramineous crops; the oat yield decreased by about 70% under 0.6% soil salinity (Zhao et al., 2013). Saline-alkali stress is therefore still an important limiting factor for the growth, development, yield, and quality of oats (Sun et al., 2010). Previous studies on salt or saltine-alkali tolerance of oats have focused on germplasm resource screening, and physiological and biochemical responses to saline-alkali stress (Zhang et al., 2011; Luo et al., 2012; Fu et al., 2018; Hou et al., 2018; Li et al., 2018; Zhang et al., 2018). To the best of our knowledge, the molecular mechanism for *A. sativa* L. saline-alkali tolerance remains largely unknown. An improved understanding of root metabolomics responses to saline-alkali treatment is, therefore, necessary to improve crop tolerance to this type of stress.

In this study, we described the metabolomics of BY and YZY seedling roots under complex saline-alkali stress using ultra-performance liquid chromatography–mass spectrometry (UPLC-MS/MS) analyses. The goals of this study were to (1) investigate the differences in growth and root metabolomics of oats under complex saline-alkali stress and (2) determine the differences in the saline-alkali tolerance mechanisms among tolerant and sensitive cultivars. This was a supplement to research on the mechanism of plant’s saline-alkali tolerance and provided theoretical support for the innovation of saline-alkali tolerant germplasm resources.

## Materials and Methods

### Preparation of Saline-Alkali Solutions

To simulate the saline-alkali composition and content of the soil in the central and western regions of Heilongjiang Province (Yin et al., 2017), which is one of the main oat producing areas in China, two neutral salts and two alkaline salts were mixed in a 2:1:2:1 molar ratio (NaHCO_3_: Na_2_CO_3_: NaCl: Na_2_SO_4_), resulting in total ion concentrations of Na^+^, Cl^–^, HCO_3_^–^, CO_3_^2–^, and SO_4_^2–^ of 150, 37.5, 37.5, 18.75, and 18.75 mM, respectively, with a pH of 9.8. The test design was conducted according to the method of Shi and Wang (2004), with appropriate adjustments.

### Plant Materials and Cultivation Conditions

The seeds of two oat cultivars of BY and YZY were obtained from the oat Research Laboratory of Heilongjiang Bayi Agricultural University. The oat seeds were sand cultured in a rectangular plastic culture bowl (10 cm × 10 cm) under a 25/20°C (day/night), photoperiod 12/12 h (day/night), and 70% relative humidity. Hoagland nutrient solution (20 mL bowl^–1^) was irrigated and replaced every 2 days until the first leaf of the seedlings emerged (i.e., tillering stage). Then, the control seedlings were irrigated with 20 mL of distilled water (0 mM Na^+^, pH 6.8), instead of Hoagland nutrient solution, and the treatment seedlings were irrigated with 20 mL of saline-alkali mixed solution (150 mM Na^+^, pH 9.8) instead of Hoagland nutrient solution. Each treatment contained three replicates. After 12 days of saline-alkali stress treatment, a total of 120 mL of saline-alkali solution was used for irrigation, then, the oat seedlings were ready for analysis.

### Growth Performance and Physiological Indicators

Cultured seedlings were removed and rinsed, then, the plant height, root length, and accumulation of dry matter in the shoots and its root tissues were measured, and each measurement was conducted on 10 plants and was repeated three times. The soluble protein content was determined using the Bradford method, the soluble sugar content was determined using the anthrone colorimetric method, the proline content was determined using the acidic ninhydrin colorimetry method (Gao, 2006), and the content of betaine was determined using Reinecke’s salt colorimetric method (Wang, 2006). The malondialdehyde (MDA) content was determined using the thiobarbituric acid method, the hydrogen peroxide (H_2_O_2_) content was determined using the titanium sulfate method, and the ${\text{O}}_{2}^{\cdot -}$ content was determined using the hydroxylamine method (Gao, 2006). The SOD activity was determined using nitro blue tetrazolium photo-reduction, peroxidase (POD) activity was determined using guaiacol colorimetry, CAT activity was determined using the hydrogen peroxide method, and APX was determined according to the principle that H_2_O_2_ reduces the content of ascorbic acid (Zhang and Qu, 2003).

### Metabolites of Oat Root Extracts

#### Sampling Methods

The oat seedlings were rinsed 2–3 times with phosphate-buffered saline (PBS) buffer (pH 7, 1 mol L^–^), and the oat roots were then transferred to a cryotube, frozen with liquid nitrogen, and stored at −80°C for further metabolomics analyses. Three biological replicates were performed per treatment. The BY and YZY were labeled as CKby and CKyzy, and XPby and XPyzy for the control and salt-alkali treatments, respectively.

#### Sample Preparation

The freeze-dried oat root sample was crushed by using a grinder (MM400; Retsch, Germany) for 1.5 min at 30 Hz. Then, 100 mg of powder was weighed and extracted overnight in a refrigerator at 4°C with 1 mL of extract solution (methanol: water = 7: 3). The sample was vortexed three times during this period to increase the extraction efficiency. After centrifugation at 10,000 rpm for 10 min, the supernatant was collected, filtered with a microporous filter (0.22 μm), and stored in the injection bottle for further LC–MS/MS analysis. The quality control sample was prepared by mixing equal amounts of the four groups of oat root extracts from different treatments. One quality control sample, which was prepared by mixing an equal aliquot of the supernatants from all samples, was used and replicated three times to examine the repeatability of the entire analysis process.

#### LC–MS/MS Analysis

The ultra-performance liquid chromatography (UPLC) separation was conducted using a Shim-pack UFLC CBM30A System (Shimadzu, Kyoto, Japan), equipped with a UPLC HSS T3 C18 (1.8 μm, 2.1 mm × 100 mm; Waters, Milford, MA, United States). The mobile phase was composed of ultrapure water (with 0.04% acetic acid) and acetonitrile (with 0.04% acetic acid). An elution gradient with a water/acetonitrile ratio of 95:5 (v/v) at 0 min, 5:95 (v/v) at 11 min, 5:95 (v/v) at 12 min, 95:5 (v/v) 12.1 min, and 95:5 (v/v) at 15 min was performed. The flow rate was set to 0.4 mL min^–1^, with a column temperature at 40°C and injection volume at 2 μl.

The Biosystems 6500 QTRAP was used to acquire MS/MS spectra of the metabolites. After the sample was chromatographically separated, it was subjected to MS for analysis, and the eluent was alternately connected to electrospray ionization (ESI)-triple quadrupole (QQQ). The MS conditions for a multiple reaction monitoring (MRM) test was set as follows: the electrospray ion source temperature 500°C, the ion spray voltage as 5500 or −4500 V in the positive or negative modes, respectively; the gas I as 55 psi gas II as 60 psi, the curtain gas as 25 psi, and the collision-activated dissociation as high. The QQQ scans were acquired as MRM experiments with the collision gas (nitrogen) set to 5 psi, and each ion pair was scanned according to the optimized declustering potential (DP) and collision energy (CE; Chen et al., 2013). The Analyst 1.6.3 software (AB Sciex, Waltham, MA, United States) was used to continuously evaluate the full scan survey of MS/MS data, as it was collected and triggered the acquisition of MS/MS spectra (Supplementary Figure 1).

### Data Processing and Analysis

#### Qualitative and Quantitative of Metabolites

Metabolite identification was conducted based on the metabolite information MWDB (Metware Database built by BioMarker) and public databases, such as MassBank, KNAPSAcK, HMDB, and METLIN. A quantitative analysis of metabolites used MRM with QQQ mass spectrometry. Data were processed using Analyst 1.6.3 software, and peak areas were integrated and corrected by the MultiaQuant software (Fraga et al., 2010; Supplementary Figure 1).

#### Data Preprocessing and Annotation

Metabolite data were log_10_-transformed for statistical analysis to improve the normality and were normalized. Unsupervised pattern recognition principal component analysis (PCA) on normalized data was performed using the R software, version 3.1.1. The supervised pattern recognition orthogonal partial least squares discriminant analysis (OPLS-DA) was tested for all samples, and the R (3.3.2) package *ropls* was adopted to analyze the differences between the samples and to identify differential metabolites. The OPLS-DA model was used with variable importance in the projection (VIP) values (VIP > 1) combined with FC (fold change) of metabolites values (FC > 2) to identify differential metabolites. The Kyoto Encyclopedia of Genes and Genomes (KEGG) database was used to perform functional annotations on differential metabolites. Based on the annotation results, a KEGG pathway enrichment analysis was performed.

#### Statistical Data Analysis

The effects of different concentrations of saline-alkali stress on the physiological parameters of roots were analyzed by one-way ANOVA with the Duncan’s method for multiple comparisons at significance levels of *P* ≤ 0.05 and 0.01. All statistical analyses of experimental data were performed using the SPSS software v.20.0 (SPSS, Chicago, IL, United States).

## Results

### The Growth Phenotype Assay

**FIGURE 1:**
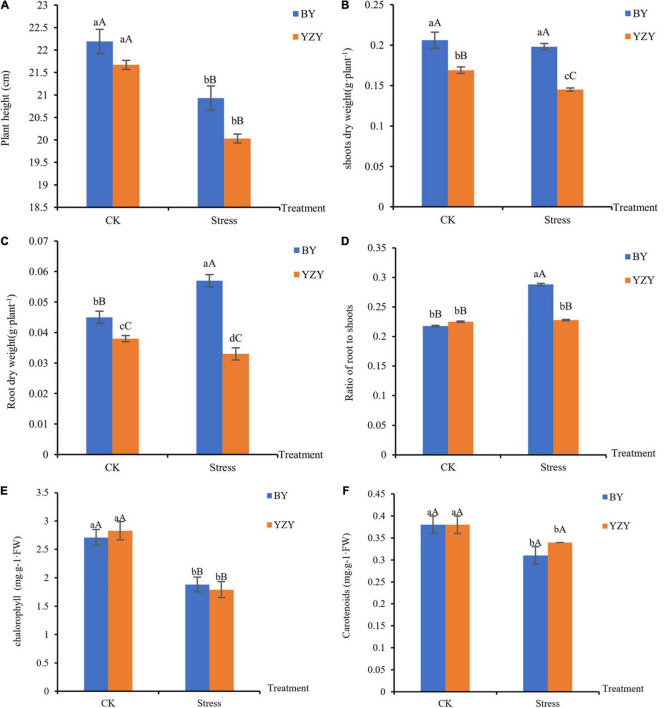
Oat seedlings growth phenotypes of Baiyan7 (BY) and Yizhangyan4 (YZY) during saline-alkali stress. **(A)** Plant height (cm), **(B)** shoots dry weight (g plant^–1^), **(C)** root dry weight (g plant^–1^), **(D)** ratio of root weight to shoot weight, **(E)** chlorophyll content in the leaves (mg⋅g^–1^⋅FW), and **(F)** carotenoids content in the leaves (mg⋅g^–1^⋅FW). Different lowercase letters indicate significant differences among the treatments (*P* < 0.05) and different capital letters indicate significant differences (*P* < 0.01).

Saline-alkali stress lasting for 12 days caused a decrease in plant heights by 5.68% (BY) and 7.57% (YZY), as compared to the respective controls; but there was no significant difference among cultivars under the same treatment (Figure 1A). For YZY, saline-alkali stress induced a significant decrease in the biomass of shoots and roots by 14.20 and 13.16%, respectively, in the stressed group when compared with its respective controls. For BY, saline-alkali stress did not induce a significant change in the biomass of shoots and resulted in a significant increase of root dry weight by 26.67% (Figures 1B,C). Under saline-alkali stress, shoots dry weight, root dry weight, and the ratio of roots to shoots in YZY decreased significantly by 26.77, 42.11, and 20.83%, respectively, compared to BY (Figures 1B–D).

The plant height of the two oat cultivars was significantly decreased (*P* < 0.01) after saline-alkali stress, but there was no significant difference among cultivars under the same treatment (Figure 1A). For BY, saline-alkali stress did not significantly reduce the biomass of shoots and roots, but it instead induced a significant increase (*P* < 0.01) in root’s dry weight and the ratio of roots to shoots ratio (*P* < 0.01; Figures 1B–D). However, the saline-alkali stress resulted in a significant decrease (*P* < 0.05) in above-ground and below-ground biomasses in YZY (Figures 1B,C). These results showed that there were differences in growth phenotypes among cultivars after saline-alkali stress. At the same time, the chlorophyll content in leaves decreased during saline-alkali stress (*P* < 0.01; Figure 1E).

### Reactive Oxygen Species and Malondialdehyde Accumulation

Under saline-alkali stress, distinct growth phenotype differences suggested that oat root cells were likely to face a serious threat. To verify this hypothesis, related physiological and biochemical analyses were conducted.

**FIGURE 2:**
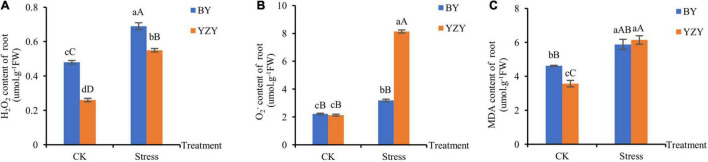
Accumulation of H_2_O_2_, ${\text{O}}_{2}^{\cdot -},$ and malondialdehyde (MDA) in roots under saline-alkali stress. **(A)** H_2_O_2_, **(B)** ${\text{O}}_{2}^{\cdot -},$ and **(C)** MDA contents in roots under control and saline-alkali stress. FW, fresh weight.

The significantly higher ROS content, which was induced by saline-alkali stress, was observed in the roots of both cultivars. The H_2_O_2_ concentration (0.69 μmol g^–1^ FW) in the treated roots of BY was the highest, which was significantly higher than that of YZY roots (Figure 2A). The roots of YZY had the highest ${\text{O}}_{2}^{\cdot -}$ concentration (8.14 μmol g^–1^ FW), which was significantly higher than that of BY roots (Figure 2B). Compared with the control, increases in H_2_O_2_ and ${\text{O}}_{2}^{\cdot -}$ were the largest in YZY roots, which was significantly increased by 111.54 and 282.16%, respectively. The MDA, which reflects cell membrane damage, increased significantly by 27.27 and 71.99% in BY and YZY roots, respectively. There was no significant difference in the content of MDA in roots between the two cultivars after treatment (Figure 2C).

### Antioxidant Enzyme Activities of Oat Seedlings Roots

**FIGURE 3:**
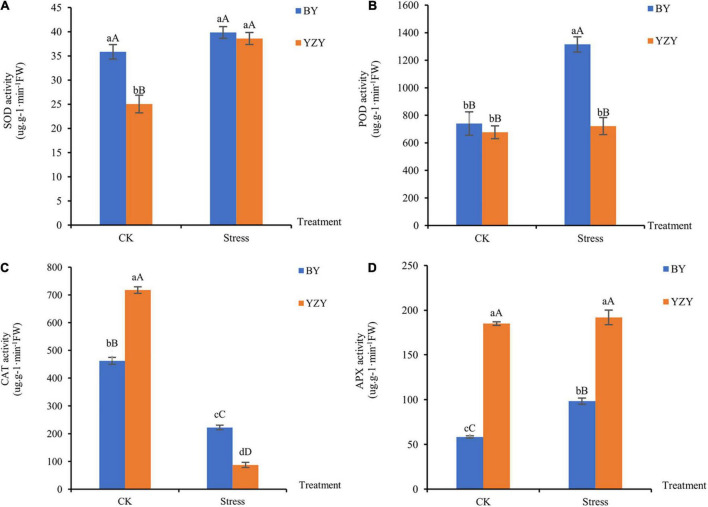
Antioxidant enzyme activity of oat seedling roots during CK and saline-alkali stress conditions. **(A)** Activity of superoxide dismutase, **(B)** activity of peroxidase, **(C)** activity of catalase, and **(D)** activity of ascorbate peroxidase. FW, fresh weight; DW, dry weight.

Saline-alkali stress-induced changes in the antioxidant enzyme activities of oat roots. After saline-alkali stress, the activity of SOD in YZY roots increased significantly by 54.15% (*P* < 0.01; Figure 3A); the activities of POD and APX in BY roots were significantly increased by 77.70 and 68.58% compared to control, respectively (*P* < 0.01; Figures 3B,D). The activity of CAT in the roots of both cultivars decreased significantly (*P* < 0.01; Figure 3C). Under saline-alkali stress, the activities of POD and CAT in BY roots were significantly higher than those in YZY roots; however, the APX activity was significantly lower than that of YZY root.

### Osmo-Regulated Substance Changes of Oat Seedling Roots

**FIGURE 4:**
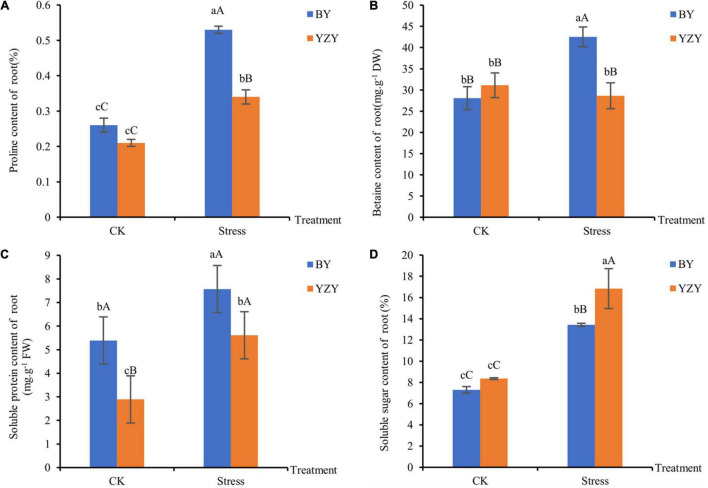
Osmoregulation substances of oat seedling roots under CK and saline-alkali stress conditions. **(A)** Proline content of BY and YZY roots under saline-alkali stress treatment and CK conditions. **(B)** Betaine content, **(C)** soluble protein content, and **(D)** soluble sugar content. FW, fresh weight; DW, dry weight.

The main osmoregulation substances such as proline, betaine, soluble sugar, and soluble protein were significantly affected by saline-alkali stress. The results showed that the contents of proline, soluble protein, and soluble sugar in the roots of both cultivars were significantly higher than those of the control (*P* < 0.05). Moreover, the content of betaine was significantly increased in BY roots (*P* < 0.01); but there was no significant change in YZY roots (Figure 4). After saline-alkali stress, the contents of proline, betaine, and soluble protein but not soluble sugar in the roots of BY, were significantly higher than those in the roots of YZY (*P* < 0.05), which increased by 55.88, 48.55, and 34.94%, respectively (Figures 4A–C). The soluble sugar content in the roots of BY was significantly increased by saline-alkali stress, but it was significantly lower than that of YZY after stress (Figure 4D).

### Principal Component Analysis of Oat Root Metabolites Under Saline-Alkali Stress

The results of the analysis of main osmoregulation substances showed that there were significant differences in osmotic adjustment responses to saline-alkali stress between the two cultivars. To further validate these results, a metabonomic analysis was conducted.

**FIGURE 5:**
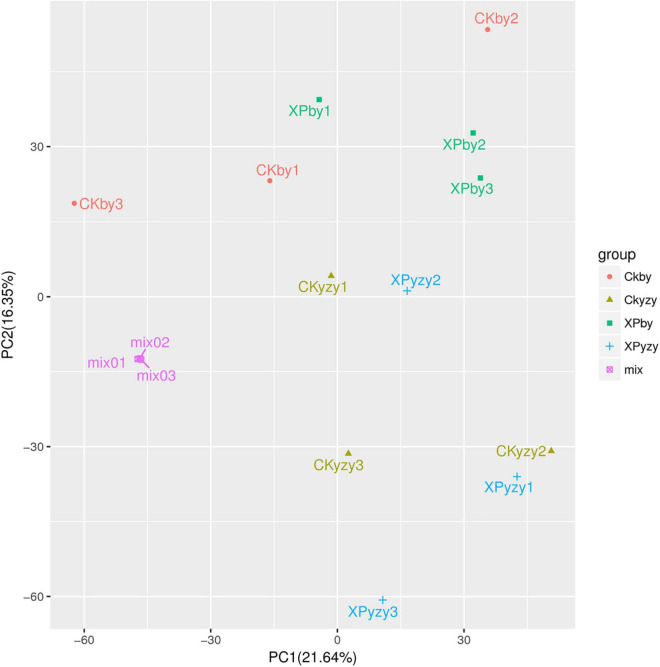
Principal component analysis score plots of metabolic profiles in oat roots. CKby and CKyzy represent the control treatment of Baiyan7 (BY) and Yizhangyan4 (YZY), respectively; XPby and XPyzy represents the saline-alkali stress treatment of BY and YZY, respectively.

**FIGURE 6:**
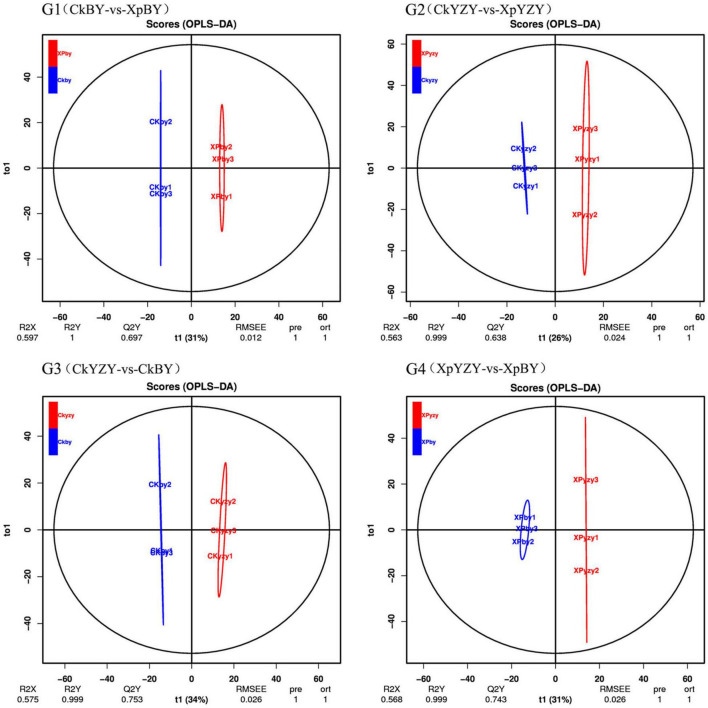
The orthogonal partial least squares discriminant analysis model score plot of metabolic profiles in oat roots.

The chromatograms of oat seedling roots were obtained using LC–MS/MS analysis (Supplementary Figure 1). There were significant differences between the peaks. The score plots of PCA results (Figure 5) showed that the control and treatment clusters were separated by the first principal (PC1), which accounted for 21.64% of the total variance, and the BY and YZY clusters were separated by the second principal (PC2), which accounted for 16.53% of the total variance. The separation performance of PCA analysis was not obvious. Given the complexity of the data, OPLS-DA was performed to accurately analyze the differences between the samples within each group (Figure 6). The score plots of the OPLS-DA results showed clear separations, and the Q2, which represented the model predictability, was greater than 0.5, indicating the effectiveness of the model. These results indicated that the samples within each group were significantly different in their metabolic levels, and the differential metabolites could be screened according to their VIP values.

### Differential Metabolite Analysis

**FIGURE 7:**
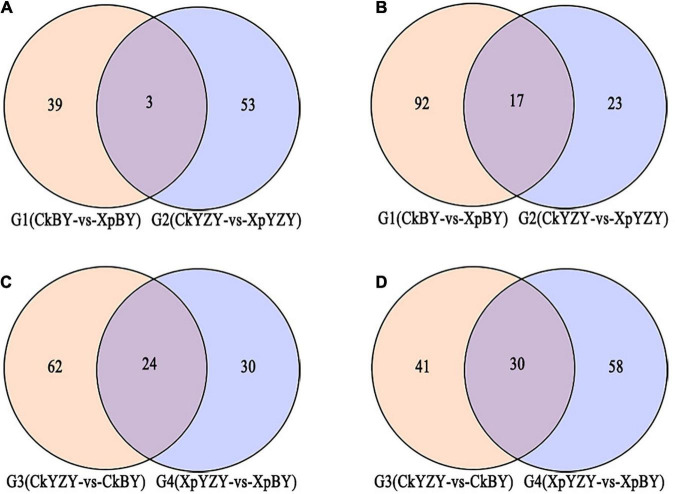
Venn diagram of different metabolites between groups. **(A,C)** Represent upregulated metabolites and **(B,D)** represent downregulated metabolites.

In BY and YZY roots, 752 metabolites were identified by LC–MS/MS. According to the OPLS-DA model, metabolites with VIP > 1 and FC > 2 were determined as differentially abundant metabolites (DAMs). After saline-alkali stress, there were 42 upregulated DAMs and 109 downregulated DAMs in BY roots, and 56 upregulated DAMs and 40 DAMs in YZY roots (Table 1 and Supplementary Table 1); while there were only 20 common DAMs, including 3 upregulated DAMs and 17 downregulated DAMs in groups G_1_ and G_2_ (Figures 7A,B). Compared with YZY, there were 86 upregulated DAMs and 71 downregulated DAMs in BY roots before treatment; and 54 upregulated DAMs and 88 downregulated DAMs in BY roots during stress (Table 1 and Supplementary Table 1); there were only 54 common DAMs, including 24 upregulated DAMs and 30 downregulated DAMs in G_3_ and G_4_ (Figures 7C,D), which indicated that saline-alkali stress led to changes in metabolites and that there were differences in metabolic profiles between cultivars. We then focused on differential metabolites induced by saline-alkali stress in G1 (CkBY-vs-XpBY) and G2 (CkYZY-vs-XpYZY).

**TABLE 1:** Differential metabolites between different treatment groups of oat roots.

Group name	Differential metabolite	Down	Up	Differential metabolite in pathway
G1 (CkBY-vs-XpBY)	151	109	42	36
G2 (CkYZY-vs-XpYZY)	96	40	56	26
G3 (CkYZY-vs-CkBY)	157	71	86	46
G4 (XpYZY-vs-XpBY)	142	88	54	43

Difference significance analysis was conducted for DAMs induced by saline-alkali stress. Compared with the control, the significantly (*P* < 0.05) up-accumulated DAMs in BY roots were mainly amino acids and amino acid derivatives, phenylpropanoid compounds, including betaine (14.27-fold), 3-*O*-acetyl pinobanksin (11.00-fold), 1-*O*-feruloyl quinate (1.80-fold), *N*-acetyl-L-arginine (1.45-fold), L-cysteine (1.30-fold), proline (1.24-fold), *p*-coumaryl alcohol (1.29-fold), 3-methyl-1-pentanol (1.14-fold), pantothenol (1.12-fold), and 8-methoxy psoralen (1.09-fold). In contrast, the significantly (*P* < 0.05) up-accumulated DAMs in YZY roots were mainly organic acids, phenylpropanoid, and flavonoid compounds, including kaempferol 3-*O*-glucoside (2.80-fold), kaempferol 3-*O*-galactoside (2.34-fold), citric acid monohydrate (1.44-fold), *N*-feruloyl putrescine (1.36-fold), pantothenol (1.26-fold), citric acid (1.19-fold), 1-*O*-beta-D-glucopyranosyl sinapate (1.11-fold), and *cis*-aconitic acid (1.08-fold). Some lipids, phenylpropanoids, flavonoids, and other compounds were significantly (*P* < 0.05) down-accumulated metabolites, but there were differences in compound types and downregulation levels in the two cultivars (Table 2).

**TABLE 2:** The significant differential abundant metabolites in roots of Baiyan7 (BY) or Yizhangyan4 (YZY) after saline-alkali stress.

Compounds		BY roots			YZY roots		
		log_2_FC	VIP	Regulated	log_2_FC	VIP	Regulated
Amino acids and derivatives	L-Cysteine	1.30	1.76	↑^A^	0.41	1.74	–
	Betaine	14.27	1.72	↑^a^	0.18	0.20	–
	Proline	1.24	1.65	↑^a^	0.48	1.24	–
	*N*-acetyl-L-arginine	1.45	1.65	↑^a^	2.61	1.59	↑
	*N*-feruloyl putrescine	0.56	1.09	–	1.36	1.76	↑^a^
	*N*,*N*-dimethylglycine	−0.43	0.87	–	−1.11	1.78	↓^a^
Organic acids	Citric acid monohydrate	0.30	0.54	–	1.44	1.90	↑^A^
	Citric acid	0.26	0.57	–	1.19	1.84	↑^A^
	*Cis*-Aconitic acid	0.27	0.95	–	1.08	1.80	↑^a^
Lipids and derivatives	MAG (18:1) isomer2	−2.54	1.70	↓^A^	−1.09	1.01	↓^a^
	MAG (18:1) isomer1	−2.48	1.65	↓^A^	−1.13	1.01	↓^a^
	LysoPE 14:0	−2.11	1.56	↓^a^	−1.29	1.32	↓^a^
	LysoPC 16:1 (2n isomer)	−1.41	1.50	↓^a^	−1.26	1.26	↓^a^
	MAG (18:3) isomer2	−1.65	1.67	↓^A^	−0.43	0.46	–
	MAG (18:3) isomer3	−1.85	1.65	↓^a^	−0.52	0.52	–
	DGMG (18:1)	−1.98	1.61	↓^a^	−0.46	0.39	–
	DGMG (18:2) isomer2	−2.57	1.61	↓^a^	−0.52	0.39	–
	MAG (18:3) isomer5	−2.10	1.58	↓^a^	−0.71	0.57	–
	Ureido isobutyric acid	−0.20	0.16	–	−10.77	1.93	↓^A^
Phenylpropanoids	1-*O*-Feruloyl quinate	1.80	1.63	↑^a^	0.70	1.44	–
	*p*-Coumaryl alcohol	1.29	1.62	↑^a^	0.84	0.89	–
	8-Methoxy psoralen	1.09	1.65	↑^a^	0.41	0.61	–
	4-Methoxy cinnamic acid	−1.82	1.61	↓^a^	0.79	0.81	–
	Osthole	−1.81	1.59	↓^a^	−0.83	0.80	–
	*trans*-4-Hydroxycinnamic acid Methyl Ester	−1.79	1.61	↓^a^	0.78	0.82	–
	Methyl *p*-coumarate	−1.68	1.59	↓^a^	0.78	0.84	–
	Methyl ferulate	−1.60	1.50	↓^a^	0.65	0.59	–
	3,4-Dimethoxycinnamic acid	−1.60	1.50	↓^a^	0.66	0.60	–
	1-*O*-Caffeoyl quinate	−1.30	1.54	↓^a^	−0.43	0.65	–
	Caftaric acid	−1.02	1.50	↓^a^	0.77	1.35	–
	1-*O*-beta-D-Glucopyranosyl sinapate	0.63	0.95	–	1.11	1.83	↑^a^
	3,4,5-Trimethoxycinnamic acid	−0.01	0.02	–	−1.25	1.88	↓^a^
Flavonoids	3-*O*-Acetylpinobanksin	11.00	1.75	↑^A^	−0.26	0.21	
	Dihydroquercetin	−1.01	1.69	↓^A^	−0.24	1.27	–
	Glabridin	−1.89	1.63	↓^a^	−0.41	0.32	–
	Kaempferol 3-*O*-glucoside (astragalin)	−3.24	0.74	–	2.80	1.77	↑^a^
	Kaempferol 3-*O*-galactoside (trifolin)	−3.42	0.73	–	2.34	1.65	↑^a^
	Spinosin	0.42	0.46	–	−11.19	1.94	↓^A^
	Liquiritigenin	0.07	0.08	–	−2.49	1.74	↓^a^
	Apigenin *O*-hexosyl-*O*-pentoside	−0.49	1.69	–	−1.17	1.69	↓^a^
Others	Pantothenol	1.12	1.65	↑^A^	1.26	1.80	↑^a^
	3-Methyl-1-pentanol	1.14	1.60	↑*^a^*	0.41	1.12	–
	*N*-acetyl glucosamine 1-phosphate	−1.06	1.54	↓^a^	−0.49	1.24	–
	6-Gingerol	−1.09	1.09	↓^a^	−2.60	1.77	↓^a^
	Gallic acid	0.79	0.38	–	−13.94	1.94	↓^A^

*The fold’s change was calculated by the formula: log_2_^(saline–alkali/control)^; the ↑^a^ and ↑^A^ indicate significant and highly significant decreases, the ↓^a^ and ↓^A^ indicate significant and highly significant decreases, respectively.*

For BY roots, there were 42 upregulated DAMs and 109 downregulated DAMs in the saline-alkali stress-exposed samples, when compared to the control. A total of 151 DAMs included 5 sugars (such as trehalose 6-P, *N*-acetylglucosamine 1-P, and phytocassane, etc.), 35 organic acids and derivatives (such as fumaric acid, caffeic acid, cinnamate, and *trans*-cinnamate, etc.), 12 amino acids and derivatives (such as proline, cysteine, and methionine, etc.), 33 lipids [most of which were monoacylglycerol (MAG) and its isomer, lysoPC and its isomer, and scope and its isomer, etc.], 11 phenylpropanoids (including 4 monolignols and 7 coumarins, etc.), 14 flavonoids (such as taxifolin, glabridin, 3-*O*-acetyl pinobanksin, and luteolin, etc.), 24 glycosides (most of them were formed by flavonoids and sugars; most of which were downregulated), 5 vitamins and cofactors (such as α-tocotrienol, pantothenol, and thiamine, etc.), 6 alkaloids, 2 peptides, 6-gingerol, phytol, and 4-hydroxy benzaldehyde (Table 1 and Supplementary Table 1). Among them, the DAMs with larger multiples mainly included fumaric acid (16.38-fold), betaine (14.27-fold), rosmarinic acid (13.24-fold), L-isoleucine (12.98-fold), imperatorin (−13.92-fold), and α-tocotrienol (−12.20-fold) (Figure 7). The significantly (*P* < 0.05) increased DAMs were mainly amino acids and their derivatives, phenylpropanoid compounds, including betaine, 3-*O*-acetyl pinobanksin, 1-*O*-feruloyl quinate, *N*-acetyl-L-arginine, cysteine, proline, *p*-coumaryl alcohol, 3-methyl-1-pentanol pantothenol, and 8-methoxypsoralen. The significantly (*P* < 0.05) decreased DAMs were mainly lipids and organic acids (Table 2).

Compared to the control, there were 56 upregulated DAMs and 40 downregulated DAMs induced by saline-alkali stress in YZY roots. A total of 151 DAMs included 8 sugars (including upregulated maltotetraose, galactinol, mannitol, melezitose, panose, and downregulated sucrose and deoxyribose 5-P), 24 organic acids and derivatives (such as citric acid, *cis*-aconitic acid, citramalate, ureidoisobutyric acid, deoxycytidine, 2′-deoxyinosine, and *trans*-citridic acid), 4 amino acids and derivatives, 12 lipids, three phenylpropanoids, 10 flavonoids (such as liquiritigenin, tangeretin, tricin, and catechin), 19 glycosides (most of them were formed by flavonoids and sugar, and most of which were upregulated), two vitamins and cofactors (the reduced form of glutathione and pantothenol), 6 alkaloids, 3 peptides (*N*′-*p*-coumaroyl putrescine, *N-p*-coumaroyl putrescine, and *N*-feruloyl putrescine), 6-gingerol, gallic acid, ginkgolide A, and chlorpyrifos (Table 1 and Supplementary Table 1). Among them, the more complex DAMs included *C*-hexosyl-chrysoeriol *O*-hexoside (15.5-fold), D (+)-melezitose *O*-rhamnoside (10.21-fold), maltotetraose (4.08-fold), melezitose (3.20-fold), gallic acid (−13.94-fold), deoxyribose 5-P (−11.67-fold), spinosin (−11.19-fold), rosmarinic acid (9.68-fold), and catechin (−2.86-fold) (Figure 7). The significantly (*P* < 0.05) increased DAMs included two metabolites related to the TCA cycle (citric acid and *cis*-aconitic acid), three glycosides, pantothenol, *N*-feruloyl putrescine, and citric acid monohydrate, and the decreased DAMs included gallic acid, spinosyn, ureidoisobutyric acid, and dimethylglycine (Table 2).

### Functional Annotation and Enrichment Analysis of Differentially Abundant Metabolites in the Kyoto Encyclopedia of Genes and Genomes

The results of metabolic pathway annotation analyses identified 36 DAMs from BY and 26 DAMs from YZY of the corresponding metabolic pathways (Table 1). In BY roots, DAMs showing significantly increased accumulation levels under saline-alkali stress were mainly concentrated in amino acids and their derivatives (such as proline, *N*-acetyl-L-arginine, and L-cysteine) in the biosynthesis of amino acids pathway, in the serine and threonine metabolism pathway, and phenylpropanoids and their derivatives (such as *p*-coumaryl alcohol and 1-*O*-feruloyl quinate) in the phenylpropanoid biosynthesis pathway. The DAMs, showing significantly reduced accumulation levels under saline-alkali stress, were mainly concentrated in lipids and their derivatives [such as MAG, monogalactosyl diacylglycerol (DGMG), lysoPE, and lysoPC] in the glycerolipid metabolism pathway and the lipid metabolism pathway, phenylpropanoids and their derivatives in the phenylpropanoid biosynthesis pathway (such as 4-methoxy cinnamic acid, osthenol, caffeic acid, methyl *p*-coumarate, *trans*-4-hydroxycinnamic acid methyl ester, 3,4-dimethoxycinnamic acid, ethyl ferulate, and 1-*O*-caffeoyl quinate imperatorin), and flavonoid compounds (dihydroquercetin and glabridin) (Table 2 and Figure 9). In YZY roots, significantly upregulated DAMs found under saline-alkali stress were mainly concentrated in the TCA cycle and its intermediate metabolites (such as citric acid, citric acid monohydrate, and *cis*-aconitic acid), and flavonol compounds in the flavone and flavonol biosynthesis pathways (such as kaempferol 3-*O*-glucoside, and kaempferol 3-*O*-galactoside). The significantly downregulated DAMs mainly included some flavonoid compounds (such as spinosyn, liquiritigenin, and apigenin *O*-hexosyl-*O*-pentoside) (Table 2 and Figure 9).

Under saline-alkali stress, the TCA cycle of BY and YZY seeding roots was stimulated in the roots of BY and YZY, and the intermediates, such as citrate, *cis*-aconitate, succinate, fumarate, and malate, were increased. Compared with BY, the TCA cycle pathway components were enhanced by saline-alkali stress in YZY roots. The amino acid metabolism and related metabolism pathways were significantly altered in BY and YZY; in the arginine and proline metabolism pathway, proline in BY roots was upregulated, while a large amount of *N*-feruloyl putrescine, *N-p*-coumaroyl putrescine, and *N*′-*p*-coumaroyl putrescine accumulated in YZY roots. Compared with BY, the sugar and alcohol metabolisms in YZY roots were increased, as were melibiose, sucrose, galactinol, glucose-1P, glucose-6P, trehalose-6P, fructose-6P, maltotetraose, melezitose, and panose accumulation. For these metabolites, the increases were greater in YZY roots (Figure 9). In the phenylpropanoid biosynthesis pathway, saline-alkali stress induced the decrease of caffeate, methyl ferulate, methyl *p*-coumarate, *trans*-4-hydroxycinnamic acid methyl ester, 3,4-dimethoxycinnamic acid, 1-*O*-caffeoyl quinic acid, and 4-methoxycinnamic acid accumulation in BY roots, and increases of downstream products including *p*-coumaryl alcohol, coniferyl alcohol, and sinapyl alcohol. In contrast, saline-alkali stress led to the enhancement of the flavonoid biosynthesis pathway and the production of flavonoids, followed by glycosides (formed by flavonoids and sugar) accumulated in YZY roots (Figure 9 and Table 2).

## Discussion

The BY cultivar was bred by the Jilin Baicheng Academy of Agricultural Sciences (Guo et al., 2007); the YZY cultivar was bred by the Zhang Jiakou Academy of Agricultural Sciences. Both BY and YZY are the main cultivars of oats used for foods or feeds in the western Songnen Plain (Fu et al., 2018). According to the screening results (data not listed) of the experiments, the BY and YZY were identified as saline-alkali resistant and sensitive cultivars, respectively.

Saline-alkali stress severely inhibits plant growth and even leads to plant death, with seed germination and seedling growth being the most sensitive stages affected (Ibrahim, 2016). Roots are the main site of nutrient uptake and salinity perception (Bai et al., 2016), which plays an important role in plant growth and development. Roots are in direct contact with soil, and are the first perceiver and responder of soil environmental stress; roots are more sensitive than shoots, responding to alkali stress (Xu et al., 2013; Wang et al., 2021). When roots are exposed to saline-alkali stress, the root is the first to perceive the stress information, which is gradually transmitted to the aboveground parts (Fang et al., 2021).

### Inhibition of Seedling Growth and Physiological Parameters

The plant height of the two cultivars decreased significantly under saline-alkali stress, indicating that the plant growth of oats was significantly affected. Dry matter accumulation level is a comprehensive reflection of seedlings and one of the main indices of salt tolerance (Yang et al., 2009; Wang et al., 2018). A high pH stress mainly reduced the chlorophyll content (Bai et al., 2021), which caused a significant decrease in shoot and root dry weights of YZY (Figure 1). Saline-alkali stress did not have a negative impact on the BY root dry weight and even induced a significant increase in the root dry weight and root/shoot ratio. Maintaining vigorous root growth and increasing the root/shoot ratio may be a morphological adaptation for saline-alkali stress-tolerant cultivars (BY) (Wang et al., 2018). This is consistent with previous studies (Wu et al., 2009; Luo et al., 2012) and may be due to the difference in response mechanisms of different oat cultivars under saline-alkali stress, leading to differences in their growth performances.

Under saline-alkali stress, the rapid and excessive accumulation of ROS, including H_2_O_2_ and O_2_^–^ can cause damage to cell membranes through lipid peroxidation and can even cause cell death (Gill and Tuteja, 2010; Liu et al., 2020). Among the antioxidant enzymes, CAT is sensitive to saline-alkali reaction, and, APX and POD play important roles in H_2_O_2_ clearance (Liu et al., 2016). In the present study, obvious effects of saline-alkali stress on the accumulation of H_2_O_2_ and O_2_^∙–^ were observed in the roots of both cultivars (Figure 2). The MDA, which reflects increased cell membrane damage, showed no significant differences among cultivars. This indicated that saline-alkali stress caused the accumulation of ROS in oat roots, but showed no significant difference in the degree of cell membrane damage between cultivars. Similar results for oats (*Avena nude* L.) have been reported (Zhao et al., 2013; Liu et al., 2016).

Osmotic stress has been identified as one of the main threats to plants under saline conditions (Munns, 2002). To cope with osmotic stress, plants synthesize and accumulate compatible solutes, and the most seen and compatible solutes are sucrose, proline, and betaine, which can generate a significant osmotic pressure and function as osmolytes during salt stress (Munns and Tester, 2008). Different plant species and different cultivars of the same species can respond to salt-alkali stress through changes in different osmotic adjustment substances. Accumulations of proline and betaine are generally higher in salt-tolerant plant species than in sensitive species (Liu et al., 2016; Fu et al., 2018). Consistent with these results, during saline-alkali stress, the contents of proline, betaine, soluble protein, and soluble sugar in BY roots were significantly increased, and were significantly higher than those in YZY roots, except for soluble sugar. The contents of proline, soluble sugar, and soluble protein in YZY roots were significantly increased, and the levels of soluble sugar were significantly higher than those in BY roots (Figure 4). These results indicated that proline and betaine were the main osmotic regulators in BY roots, and that soluble sugar played a more positive osmotic regulation role in YZY roots.

### Sugar Metabolism Plays a Basic Function in Improving Saline-Alkali Tolerance

The regulation of sugar, induced by saline-alkali stress, was observed in the roots of oat (Figure 9 and Supplementary Table 1). This may be related to the function of sugars in plant metabolic pathways. In plants, sucrose is a product of photosynthesis and it is an important energy source for metabolic activity; it has the function of osmotic adjustment, affecting redox homeostasis and the protection of macromolecular structures (Yang and Guo, 2018). Glucose was downregulated in the roots of BY and YZY, and this may be due to glucose being utilized in various metabolic pathways. Sufficient carbon sources and energy supplies are important for the assimilation of nitrogen and the synthesis of amino acids (Jia et al., 2019). Therefore, we speculated that the lack of significant accumulation of sugars may be related to other metabolic enhancements, including the TCA cycle and amino acid metabolism. In addition, the saline-alkali stress-induced mannitol (1.85-fold), galactinol (2.17-fold), maltotetraose (4.08-fold), melibiose (2.02-fold), panose (3.09-fold), melezitose (1.85-fold), and sucrose (2.25-fold), are consistent with the physiological parameters for soluble sugars.

### Energy Metabolism Is an Active Strategy of Oat in Response to Saline-Alkali Stress

**FIGURE 8:**
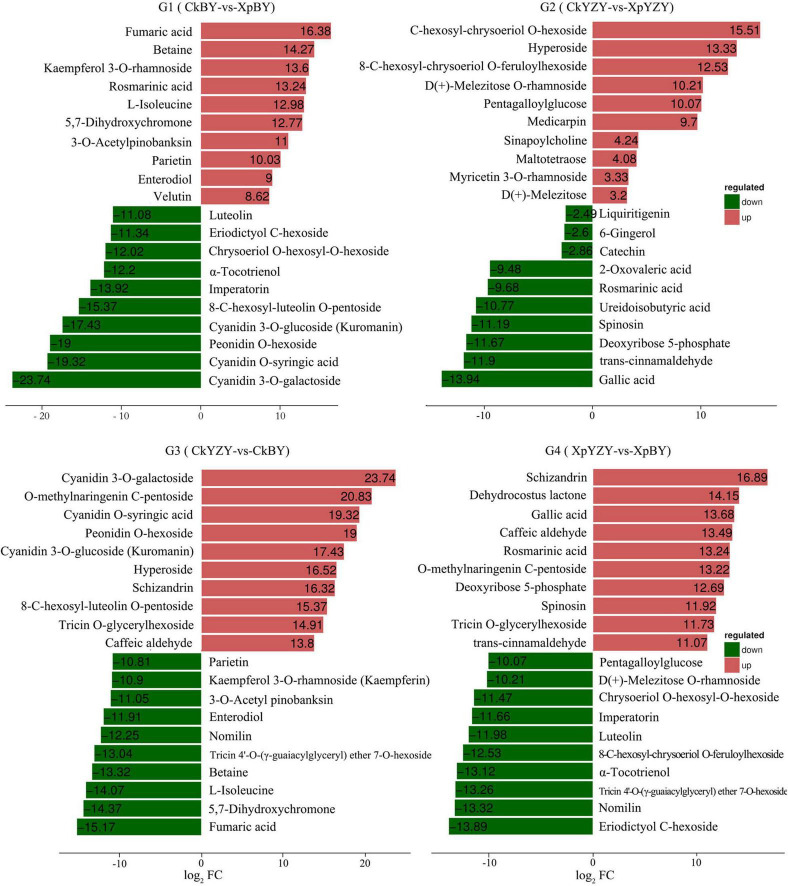
*K*-means figure of the top 10 differential metabolites (G_1_) log_2_FC (XpBY–CkBY), (G_2_) log_2_FC (XpYZY–CkYZY), (G_3_) log_2_FC (CkBY–CkYZY), and (G_2_) log_2_FC (XpBY–XpYZY). Red denotes upregulation and green denotes downregulation.

The TCA cycle is an important aerobic pathway in the final steps of the oxidation of carbohydrates and fatty acids (Wu et al., 2013) and is an important energy-producing process in plants. The TCA cycle plays an important role in resisting adverse environmental conditions, and is a central cycle in plant metabolism, giving rise to many primary and secondary metabolites including several intermediates involved in amino acid biosynthesis and nitrogen assimilation (Yang et al., 2017). The levels of *cis*-aconitic acid and citric acid in the TCA cycle were increased by saline-alkali stress in YZY roots. The intermediate metabolites of the TCA cycle, including citrate, fumarate, succinate, malate, and *cis*-aconitate, were upregulated in roots of BY and YZY (Table 2 and Figure 8), revealing that saline-alkali stress induced the enhancement of the TCA cycle. Oats could, therefore, enhance the saline-alkali tolerance of its roots by increasing energy capacity and the levels of intermediate products during saline-alkali stress, which is similar to barley (Wu et al., 2013).

### Organic Acids Maintain the Intracellular pH Under Saline-Alkali Stress

Complex saline-alkali stress further increases the pH during salt stress (Fang et al., 2021), which results in reduced ion levels in the environment surrounding the roots. A high pH leads to root cells losing their normal physiological functions (Munns and Tester, 2008). Under saline-alkali stress, plants secrete organic acids to maintain the pH and ion balance of the internal environment, which is an adaptive response to stress (Yang et al., 2017; Jia et al., 2019). Similarly, saline-alkali stress induced the accumulation of organic acids in oat roots. The plants of different families and genus (or even from the same family) have a different metabolic control mechanism for organic acids during alkali stress (Yang et al., 2007). We showed that saline-alkali stress induced the accumulation of organic acids, such as a citric acid monohydrate, *cis*-aconitic acid, citric acid, citramalate, oxalic acid, etc., in the roots of YZY. After saline-alkali stress, the organic acids accumulating in BY roots included fumaric acid, citric acid, malate, succinate, rosmarinic acid, linoleic acid, etc. The accumulation of organic acids can effectively compensate for the deficiency of inorganic anions (Wang et al., 2011). Organic acids are also involved in protein modification, nutrient uptake, signal transduction, and other physiological processes (Fang et al., 2021).

It is worth mentioning that fatty acids and their derivatives are also involved in plant resistance to abiotic stress, in addition to their roles in storing energy (Wang et al., 2021). We showed that in the roots of BY, four kinds of fatty acids, including ureidoisobutyric acid, punicic acid, octadecadien-6-ynoic acid, 16-hydroxyhexadecanoic acid, 9-hydroxy-(10E,12Z,15Z)-octadecatrienoic acid, 9,10-EODE, and 12,13-EODE, were downregulated by saline-alkali stress, which may be an effective way for BY roots to adapt to salt stress (Yang et al., 2017).

### Differences of Amino Acids and Amino Acid-Associated Metabolism Between Cultivars

**FIGURE 9:**
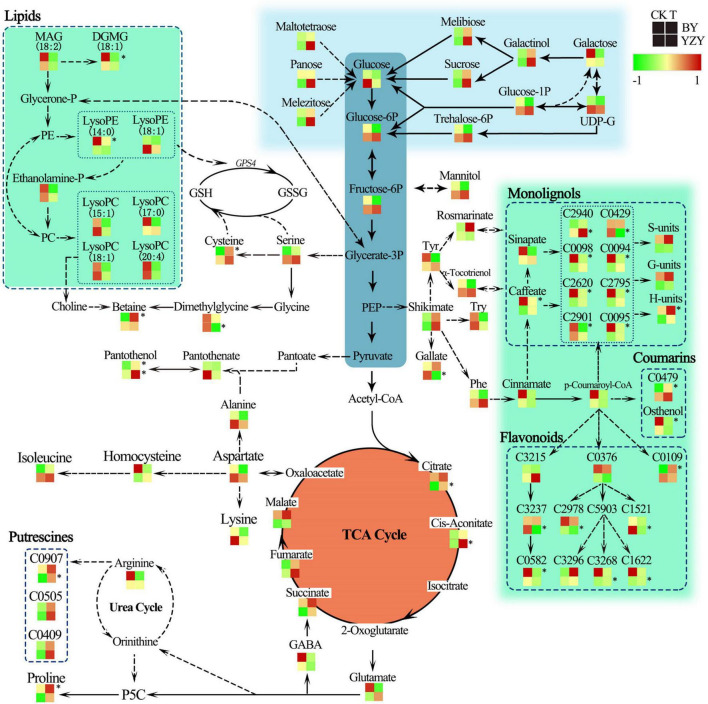
Change in metabolites of the metabolic pathways in roots of oat seedlings after 12 days saline-alkali stress treatment. Proposed metabolic network changes in oat seedlings subjected to saline-alkali stress, as obtained using orthogonal partial least squares discriminant analysis. *H*-units, *p*-coumaryl alcohol; *S*-units, sinapyl alcohol; *G*-units, coniferyl alcohol; GABA, γ-aminobutyric acid; GPS4, phospholipid-hydroperoxide glutathione peroxidase; GSH, glutathione; GSSG, glutathione disulfide; LysoPE, 1-octadecanoyl-sn-glycero-3-phosphoethanolamine; MAG, monoacylglycerol; P5C, δ-pyrroline-5-carboxylate; PE, phosphatidylethanolamine; Phe, phenylalanine; PEP, phosphoenolpyruvate trisodium salt; SAH, *S*-adenosyl-L-homocysteine; Try, tryptophan; Tyr, tyrosine; C0094, methyl *p*-coumarate; C0095, methyl ferulate; C0098, *trans*-4-hydroxycinnamic acid methyl ester; C0109, 3-*O*-acetyl pinobanksin; C0376, naringenin; C0429, 3,4,5-trimethoxycinnamic acid; C0479, 8-methoxypsoralen (xanthotoxin); C0582, glabridin; C0907, *N*-feruloyl putrescine; C1521, dihydroquercetin (taxifolin); C1622, kaempferol 3-*O*-glucoside (astragalin); C2620, 3,4-dimethoxycinnamic acid; C2795, 4-methoxycinnamic acid; C2901, 1-*O*-caffeoyl quinic acid; C2940, 1-*O*-beta-D-glucopyranosyl sinapate; C2978, apigenin *O*-hexosyl-*O*-pentoside; C3215, isoliquiritigenin; C3237, liquiritigenin; C3268, kaempferol 3-*O*-galactoside (trifolin); C3296, kaempferol 3-*O*-rhamnoside (kaempferin); C0426, pentagalloylglucose; C5903, kaempferol. *Indicate significant (*P* < 0.05).

In higher plants, amino acids accumulate in response to various stresses and have multiple functions in plant growth (Less and Galili, 2008). Amino acid accumulation occurs in response to salt stress (Mutwakil et al., 2017). The results of the current study indicated that amino acid metabolism was significantly enhanced in BY roots during saline-alkali stress, leading to the significant accumulation of proline, cysteine, isoleucine, *N*′-acetyl-L-arginine aspartate, and 4-hydroxy-L-glutamate, whereas this was not observed in YZY roots. Metabolic analyses showed that there were differences in the arginine and proline metabolism pathways between the roots of the two studied cultivars (Figure 9), including the induced significant upregulation of proline accumulation in the roots of BY, and *N*-feruloyl putrescine in the roots of YZY (*P* < 0.05). In the roots of YZY, the compounds associated with putrescine biosynthesis, such as *N*-feruloyl putrescine, *N*′-*p*-coumaroyl putrescine, and *N-p*-coumaroyl putrescine, accumulated in the roots of YZY. The *N*-feruloyl putrescine is a feruloyl-CoA conjugate of putrescine, which shares ornithine as a common precursor with proline (Mohapatra et al., 2009; Figure 9). The accumulation of polyamine putrescine is correlated with slower growth and/or necrosis, rather than being an adaptive response to salinity (Widodo et al., 2009). The elevated levels of putrescine may indicate that the roots of sensitive cultivars, such as YZY, have been extensively damaged under the experimental conditions used in the study.

The L-cysteine is used as a sulfur donor in plants and is used for multiple processes catalyzed by multiple catabolic enzymes [(Less and Galili, 2008); its level is positively correlated with the methylation index (Kim et al., 2006)]. Cysteine has a structural function in proteins, as well as playing a role as a precursor for essential biomolecules, such as glutathione (GSH), phytochelatins (PCs), *S*-adenosyl-methionine (SAM), *S*-adenosyl-L-homocysteine (SAH), and methionine (Peng and Song, 2011; Sadak et al., 2019). Our results are supported by Peng and Song (2011), who reported that the L-cysteine treatment can efficiently activate the phenylpropanoid pathway, to promote the accumulation of phenolic substances (Table 2 and Figure 9). The accumulation of isoleucine can alleviate salt stress by enhancing glycolysis (Kumar et al., 2010). In addition, these amino acids can increase the scavenging free oxygen radicals, in addition to regulating the osmotic pressure and alleviating salt stress. Moreover, amino acids serve as precursors for a large array of secondary metabolites, including pigments, alkaloids, hormones, and cell wall components (Jia et al., 2019).

Glycine betaine (GB) is synthesized from serine *via* ethanolamine, choline, and betaine aldehyde (Ashraf and Foolad, 2007), and is closely related to amino acid metabolism and lipid metabolism. Ethanolamine is essential for the synthesis of phospho-ethanolamine and phosphatidylethanolamine (PE) as well as choline and GB (Kim et al., 2006). MAG is degraded to ethanolamine *via* glycerone phosphate and PE in the glycerophospholipid metabolism pathway. In this study, the significant decrease of MAG contents in the roots of BY (Table 2 and Figure 9), including MAG (18:1) isomer2, MAG (18:1) isomer1, and MAG (18:3) isomer5, may be related to its degradation and formation of choline, which, in turn, leads to significant up-accumulation of betaine (14.27-fold). In addition to important osmotic regulation functions, the GB and proline are thought to have positive effects on enzyme and membrane integrity during stress conditions (Ashraf and Foolad, 2007). When GB is present at high levels, together with proline, it is efficient in protecting plants from oxidative stress (Annunziata et al., 2019). Furthermore, GB is only accumulated during prolonged stress, does not normally break down in plants, and can be transported easily and efficiently from older to younger plant tissues (Bray et al., 2000; Carillo et al., 2008). These results suggest that GB plays a pivotal role in protecting young leaf and root tissues of BY against saline-alkali stress, and it may be one of the reasons why BY can maintain a relatively normal growth under saline-alkali stress.

### Differences Between Cultivars of Secondary Metabolic Pathways

Generally, secondary metabolites, particularly the majority of phenolic compounds produced in the phenylpropanoid pathway, are usually a defense response to biotic or abiotic stress (Zhao et al., 2015). Compared with the control, the accumulations of secondary metabolites in oat roots, including some flavonoids and phenols, were significantly altered by saline-alkali stress (Table 2, Figure 8, and Supplementary Table 1). Considerable evidence suggests that phenylalanine, which is required for the biosynthesis of flavonoids and lignin, is an important precursor of phenylpropanoids (Santos et al., 1998). Phenylalanine accumulation was upregulated by saline-alkali stress in the roots of YZY, whereas it was downregulated in the roots of BY. This indicated that compared with YZY roots, the biosynthesis of the phenylpropanoids pathway in the roots of BY was at least partially inhibited. Analyses of metabolic pathways showed that during saline-alkali stress, phenylpropanoids and flavonoids in the roots of the two cultivars differed in metabolic pathways and metabolite accumulation levels (Figure 9).

The tolerance of plants to salinity is closely related to the formation of secondary cell walls and the deposition pattern of cellulose and lignin (Wang et al., 2016). Monolignols are synthesized from phenylalanine *via* cinnamate and *p*-coumaroyl-CoA, but this metabolic pathway can be diverted to the synthesis of flavonoids *via p*-coumaroyl-CoA (Wang, 2013). In BY roots, the increased monolignols, including coniferyl alcohol (*G*-units of lignin) and *p*-coumaryl alcohol (*H*-units of lignin) were induced by saline-alkali stress. In particular, the accumulation of *H*-units of lignin was significant (Figure 9). Increased lignin levels could contribute to the enhancement of cell wall strength, thus, maintaining and accelerating root cell growth. Unlike BY roots, lignin production in roots of YZY was not triggered by saline-alkali stress. Instead, the building blocks of phenylpropanoids were in part diverted to the production of flavonoids, suggesting suppression of root cell elongation. The tolerance of plants to salinity is closely related to the formation of secondary cell walls and the deposition pattern of cellulose and lignin (Wang et al., 2016). This may be the fundamental reason why BY roots could maintain vigorous growth under saline-alkali stress, while YZY roots were severely inhibited. The results suggested that under saline-alkali stress, the BY roots actively formed lignin units *via* the phenylpropanoid biosynthesis pathway to maintain cell structure stability and normal root growth, while the YZY roots formed flavonoids *via* the flavonoid biosynthesis pathway, and then flavonoids combined with sugars to form glycosides used for antioxidant regulation.

In addition, the panthenol in root tissues of both cultivars was significantly (*P* < 0.05) increased during stress (Table 2 and Figure 9). Pantothenol is the precursor of pantothenic acid (vitamin B5). Pantothenic acid is not an antioxidant and exerts its major function as part of CoA. It is an essential acyl group carrier co-factor required for the growth of various organisms and plays a key role in numerous steps of cellular metabolism (Awasthy et al., 2010), controlling GSH biosynthesis by regulating cellular energy levels to maintain a stable GSH level to protect against peroxidative damage (Slyshenkov et al., 2004).

## Conclusion

In summary, saline-alkali stress altered physiological, biochemical, and molecular processes, thereby affecting the growth and biomass of oat roots. The roots of oats, especially a sensitive cultivar (YZY), responded to saline-alkali stress by increasing the TCA cycle. Oat roots secreted and accumulated organic acids, which was an adaptive response to maintain pH and ion balance during saline-alkali stress. Saline-alkali stress induced the increase of amino acid metabolism in roots of tolerant cultivars (BY) and significantly increased the accumulation of osmotic substances, including proline, betaine, and L-cysteine. However, the roots of the sensitive cultivar (YZY) responded to osmotic stress by increasing the content of soluble sugars. Moreover, there were differences in the phenylpropanoid pathway between the tolerant cultivar and sensitive cultivar during saline-alkali stress. In the roots of BY, saline-alkali stress significantly induced the increase of monolignols, including coniferyl alcohol and *p*-coumaryl alcohol, which contributed to the enhancement of cell wall strength, and the maintenance and acceleration of root cell growth. However, the metabolism of phenylpropanoids was, in part, diverted to the production of flavonoids in the roots of YZY.

This is the first study that used an integrated approach to determine the possible molecular mechanisms of how *A. sativa* L. adapts to saline-alkali stress. The analyses of different metabolites of tolerant and sensitive cultivar roots provided an important theoretical basis for understanding the mechanisms of saline-alkali tolerance and increased our knowledge of plant metabolism regulation during stress.

## Data Availability Statement

The datasets presented in this study can be found in online repositories. The names of the repository/repositories and accession number(s) can be found in the article/Supplementary Material.

## Author Contributions

YG and YJ proposed the study. YG, LY, and YJ designed the experiments and secured the funding. YG, YJ, and YX conducted the field and laboratory measurements and analyzed the data. WG contributed to planning the experimental protocols and data analyses. YG and YJ wrote the manuscript, which was edited by all authors. All authors have read and agreed to the present version of the manuscript.

## Conflict of Interest

The authors declare that the research was conducted in the absence of any commercial or financial relationships that could be construed as a potential conflict of interest.

## Publisher’s Note

All claims expressed in this article are solely those of the authors and do not necessarily represent those of their affiliated organizations, or those of the publisher, the editors and the reviewers. Any product that may be evaluated in this article, or claim that may be made by its manufacturer, is not guaranteed or endorsed by the publisher.
