# Supplementary material for: Metabolic and Physiological Changes in the Roots of Two Oat Cultivars in Response to Complex Saline-Alkali Stress
Source: Front Plant Sci. 2022 Mar 29;13:835414. doi: 10.3389/fpls.2022.835414 (PMC9002314; doi:10.3389/fpls.2022.835414)

Supplementary Material

**Supplemental Table S1** Differential metabolites in roots of Baiyan7 and Yizhangyan4 induced by saline-alkali stress

| Compounds | | BY ROOTS | | | YZY ROOTS | | |
| --- | --- | --- | --- | --- | --- | --- | --- |
|  |  | log_2_FC | Regulated | VIP | log_2_FC | Regulated | VIP |
| Sugars and alcohols | Phytocassane D | 2.12 | ↑ | 1.49 | 0.35 | -- | 0.66 |
|  | 3-Methyl-1-pentanol | 1.14 | ↑* | 1.60 | 0.41 | -- | 1.12 |
|  | Trehalose 6-phosphate | -1.38 | ↓ | 1.48 | -0.08 | -- | 0.26 |
|  | Phytocassane C | -1.71 | ↓ | 1.52 | 0.19 | -- | 0.32 |
|  | N-Acetylglucosamine 1-phosphate | -1.06 | ↓ | 1.54 | -0.49 | -- | 1.24 |
|  | D-Mannitol | 0.83 | -- | 0.92 | 1.85 | ↑ | 1.44 |
|  | Galactinol | 0.93 | -- | 1.21 | 2.17 | ↑ | 1.45 |
|  | Maltotetraose | 0.54 | -- | 0.77 | 4.08 | ↑ | 1.38 |
|  | Melibiose | 0.92 | -- | 1.12 | 2.02 | ↑ | 1.37 |
|  | Panose | -0.03 | -- | 0.05 | 3.09 | ↑ | 1.45 |
|  | D (+)-Melezitose | 0.59 | -- | 0.78 | 3.20 | ↑ | 1.43 |
|  | D (+)-Sucrose | 0.99 | -- | 1.08 | 2.25 | ↑ | 1.44 |
|  | Deoxyribose 5-phosphate | 0.15 | -- | 0.18 | -11.67 | ↓ | 1.26 |
| Amino acids and derivatives | 4-Hydroxy-L-glutamic acid | 1.64 | ↑ | 1.18 | 1.50 | -- | 0.78 |
|  | L-Proline | 1.32 | ↑ | 1.53 | 0.56 | -- | 1.13 |
|  | L-Isoleucine | 12.98 | ↑ | 1.25 | 0.26 | -- | 0.28 |
|  | D-Proline | 1.24 | ↑* | 1.65 | 0.48 | -- | 1.24 |
|  | N'-Acetyl-L-arginine | 1.45 | ↑* | 1.65 | 2.61 | ↑ | 1.59 |
|  | L-Cysteine | 1.30 | ↑** | 1.76 | 0.41 | -- | 1.74 |
|  | S-(5'-Adenosy)-L-homocysteine | -1.51 | ↓ | 1.15 | 0.59 | -- | 0.87 |
|  | L-Methionine | -1.60 | ↓ | 1.18 | 0.77 | -- | 1.38 |
|  | L-Histidine | -1.11 | ↓ | 1.33 | -0.82 | -- | 1.59 |
|  | L-Tryptophan | -1.12 | ↓ | 1.13 | -0.63 | -- | 1.32 |
|  | Hexanoyl glycine | -2.22 | ↓ | 1.05 | 0.00 | -- | 0.00 |
|  | Asp-phe | -1.00 | ↓ | 1.33 | -0.13 | -- | 0.89 |
|  | 3-N-Methyl-L-histidine | 0.85 | -- | 1.65 | 1.97 | ↑ | 1.30 |
|  | 5-oxoproline | -0.70 | -- | 1.36 | -1.13 | ↓ | 1.35 |
|  | D-Alanyl-D-Alanine | -0.25 | -- | 0.95 | -1.14 | ↓ | 1.26 |
|  | N, N-Dimethylglycine | -0.43 | -- | 0.87 | -1.11 | ↓* | 1.78 |
| organic acids | Citramalate | 0.67 | -- | 1.44 | 1.87 | ↑ | 1.66 |
|  | Citric acid | 0.26 | -- | 0.57 | 1.19 | ↑* | 1.84 |
|  | Cis-Aconitic acid | 0.27 | -- | 0.95 | 1.08 | ↑* | 1.80 |
|  | Fumaric acid | 16.38 | ↑ | 1.22 | 2.08 | -- | 0.97 |
|  | trans-Cinnamate | -3.22 | ↓ | 1.37 | -0.81 | -- | 0.56 |
|  | Rosmarinic acid | 13.24 | ↑ | 1.34 | -9.68 | -- | 0.84 |
|  | Esculetin O-quinacyl esculetin O-quinic acid | -2.83 | ↓ | 1.41 | -1.52 | ↓ | 1.19 |
|  | 3-O-p-Coumaroyl quinic acid | -0.08 | no | 0.29 | 1.23 | ↑ | 1.64 |
|  | 6,7-dihydroxycoumarin 7-O-quinic acid | -2.01 | ↓ | 1.45 | -0.33 | -- | 0.21 |
|  | 1-O-Feruloyl quinic acid | 1.80 | ↑* | 1.63 | 0.70 | -- | 1.44 |
|  | Chlorogenic acid (3-O-Caffeoylquinic acid) | -1.20 | ↓ | 1.44 | -0.41 | -- | 0.60 |
|  | 1-O-Caffeoyl quinic acid | -1.30 | ↓* | 1.54 | -0.43 | -- | 0.65 |
|  | Caftaric acid（Caffeic acid） | -1.02 | ↓* | 1.50 | 0.77 | -- | 1.35 |
|  | Neochlorogenic acid (5-O-Caffeoylquinic acid) | -1.25 | ↓ | 1.39 | -0.57 | -- | 0.77 |
|  | 3,4,5-Trimethoxycinnamic acid | -0.01 | -- | 0.02 | -1.25 | ↓* | 1.88 |
|  | 4-Methoxycinnamic acid | -1.82 | ↓* | 1.61 | 0.79 | -- | 0.81 |
|  | 3,4-Dimethoxycinnamic acid | -1.60 | ↓* | 1.50 | 0.66 | -- | 0.60 |
|  | Methyl ferulate | -1.60 | ↓* | 1.50 | 0.65 | -- | 0.59 |
|  | Hydroxy-methoxycinnamate | -0.74 | -- | 0.89 | 1.46 | ↑ | 1.01 |
|  | Cinnamic acid | -3.14 | ↓ | 1.45 | -1.12 | -- | 0.63 |
|  | Methyl p-coumarate | -1.68 | ↓* | 1.59 | 0.78 | -- | 0.84 |
|  | 1-O-beta-D-Glucopyranosyl sinapate | 0.63 | -- | 0.95 | 1.11 | ↑* | 1.83 |
|  | Theaflavin-3-gallate | -4.30 | ↓ | 1.46 | 1.50 | -- | 0.69 |
|  | Gallocatechin gallate | 0.67 | -- | 0.40 | 1.84 | ↑ | 1.04 |
|  | Tricin O-glucuronic acid | 0.72 | -- | 1.37 | 1.50 | ↑ | 1.60 |
|  | Terephthalic acid | 1.23 | ↑ | 1.31 | 0.93 | -- | 1.72 |
|  | 4-Hydroxybenzoic acid | 0.95 | -- | 1.45 | 1.14 | ↑ | 1.67 |
|  | 4-Pyridoxic acid | -1.15 | ↓ | 1.19 | 0.12 | -- | 0.15 |
|  | (S)-2-(4-Aminobutanamido)-3-(1-methyl-1H-imidazol-5-yl) propanoic acid | 2.02 | ↑ | 1.30 | 0.70 | -- | 0.95 |
|  | Cytosine | -1.44 | ↓ | 1.17 | -0.05 | -- | 0.02 |
|  | Deoxyadenosine | -0.30 | -- | 0.71 | -2.02 | ↓ | 1.51 |
|  | Deoxycytidine | -0.03 | -- | 0.14 | -1.54 | ↓ | 1.39 |
|  | 2'-Deoxyinosine | -0.73 | -- | 1.20 | -1.95 | ↓ | 1.62 |
|  | Adenosine | -1.03 | ↓ | 1.46 | 0.30 | -- | 0.33 |
|  | Guanosine monophosphate（GMP） | -0.30 | -- | 0.51 | -1.13 | ↓ | 1.48 |
|  | D-Xylonic acid（D-Xylonate） | -1.27 | ↓ | 1.32 | 0.13 | -- | 0.32 |
|  | Lysine butyrate | -1.69 | ↓ | 1.34 | -1.77 | ↓ | 1.57 |
|  | Citric acid monohydrate | 0.30 | -- | 0.54 | 1.44 | ↑* | 1.90 |
|  | 4-Acetamidobutyric acid | -1.82 | ↓ | 1.22 | -0.69 | -- | 1.24 |
|  | Suberic acid | 1.10 | ↑ | 1.20 | -0.13 | -- | 0.59 |
|  | 2-Oxovaleric acid | 0.56 | -- | 0.75 | -9.48 | ↓ | 1.36 |
|  | Oxalic acid | 0.27 | -- | 0.23 | 1.71 | ↑ | 1.43 |
|  | trans-Citridic acid | 0.18 | -- | 0.65 | -1.57 | ↓ | 1.36 |
|  | 4-Hydroxy-2-oxoglutaric acid | 1.65 | ↑ | 1.26 | 0.82 | -- | 0.77 |
|  | 2-Hydroxybutanoic acid | -0.41 | -- | 0.32 | -1.70 | ↓ | 1.46 |
|  | Citraconic acid | 1.77 | ↑ | 1.15 | 2.79 | -- | 0.93 |
|  | 13-HpOTrE(r) | -1.18 | ↓ | 1.17 | 0.35 | -- | 0.63 |
|  | Ureidoisobutyric acid | -0.20 | -- | 0.16 | -10.77 | ↓* | 1.93 |
|  | Punicic acid | -1.39 | ↓ | 1.31 | -0.41 | -- | 0.93 |
|  | Octadecadien-6-ynoic acid | -1.39 | ↓ | 1.15 | -0.92 | -- | 1.55 |
|  | 16-Hydroxy hexadecanoic acid | 1.04 | ↑ | 1.28 | 0.02 | -- | 0.04 |
|  | 9-Hydroxy-(10E,12Z,15Z)-octadecatrienoic acid | -1.18 | ↓ | 1.23 | -0.72 | -- | 1.13 |
|  | 9,10-EODE | -2.36 | ↓ | 1.18 | -1.05 | ↓ | 1.68 |
|  | 12,13-EODE | -2.10 | ↓ | 1.20 | -1.22 | ↓ | 1.55 |
| Lipids | PC 16:1/14:1 | 4.07 | ↑ | 1.23 | 0.68 | -- | 0.65 |
|  | LysoPE 16:0 (2n isomer) | 1.35 | ↑ | 1.37 | 0.95 | -- | 1.72 |
|  | D-Erythronolactone | 1.68 | ↑ | 1.47 | 0.53 | -- | 1.21 |
|  | MGMG (18:2) isomer2 | -1.74 | ↓ | 1.46 | -0.59 | -- | 0.49 |
|  | LysoPC 20:4 | -1.21 | ↓ | 1.09 | -1.02 | ↓ | 1.03 |
|  | LysoPC 18:0 (2n isomer) | -1.13 | ↓ | 1.09 | -1.01 | ↓ | 1.07 |
|  | LysoPC 18:1 | -1.10 | ↓ | 1.07 | -1.06 | ↓ | 1.12 |
|  | LysoPC 16:2 (2n isomer) | -1.01 | ↓ | 1.08 | -1.12 | ↓ | 1.05 |
|  | LysoPC 15:1 | -1.17 | ↓ | 1.22 | -1.01 | -- | 0.93 |
|  | LysoPC 18:1 (2n isomer) | -1.11 | ↓ | 1.07 | -0.99 | -- | 1.05 |
|  | LysoPC 17:0 | -1.15 | ↓ | 1.07 | 0.06 | -- | 0.10 |
|  | LysoPE 14:0 (2n isomer) | -2.14 | ↓ | 1.52 | -1.21 | ↓ | 1.13 |
|  | LysoPE 18:1 (2n isomer) | -1.49 | ↓ | 1.43 | -1.23 | ↓ | 1.25 |
|  | LysoPE 18:1 | -1.24 | ↓ | 1.48 | -1.30 | ↓ | 1.40 |
|  | MAG (18:4) isomer3 | -1.85 | ↓ | 1.56 | -0.04 | -- | 0.05 |
|  | MAG (18:2) | -1.38 | ↓ | 1.50 | -0.64 | -- | 0.86 |
|  | MAG (18:3) isomer1 | -1.35 | ↓ | 1.51 | -0.12 | -- | 0.16 |
|  | MAG (18:3) isomer4 | -1.26 | ↓ | 1.49 | -0.21 | -- | 0.30 |
|  | MAG (18:4) isomer2 | -3.85 | ↓ | 1.20 | -0.95 | -- | 1.39 |
|  | 1-Eicosanol | -2.17 | ↓ | 1.15 | -0.58 | -- | 0.84 |
|  | DGMG (18:2) isomer1 | -1.57 | ↓ | 1.52 | -0.71 | -- | 0.69 |
|  | DGMG (18:2) isomer3 | -1.57 | ↓ | 1.49 | -0.79 | -- | 0.72 |
|  | D-erythro-sphinganine(Sphinganine) | -1.58 | ↓ | 1.33 | 0.84 | -- | 1.16 |
|  | D-erythro-Dihydrosphingosine | -1.77 | ↓ | 1.28 | 0.82 | -- | 1.06 |
|  | LysoPC 16:1 (2n isomer) | -1.41 | ↓* | 1.50 | -1.26 | ↓ | 1.26 |
|  | LysoPE 14:0 | -2.11 | ↓* | 1.56 | -1.29 | ↓ | 1.32 |
|  | MAG (18:3) isomer3 | -1.85 | ↓* | 1.65 | -0.52 | -- | 0.52 |
|  | MAG (18:3) isomer5 | -2.10 | ↓* | 1.58 | -0.71 | -- | 0.57 |
|  | DGMG (18:1) | -1.98 | ↓* | 1.61 | -0.46 | -- | 0.39 |
|  | DGMG (18:2) isomer2 | -2.57 | ↓* | 1.61 | -0.52 | -- | 0.39 |
|  | MAG (18:1) isomer2 | -2.54 | ↓** | 1.70 | -1.09 | ↓ | 1.01 |
|  | MAG (18:1) isomer1 | -2.48 | ↓** | 1.65 | -1.13 | ↓ | 1.01 |
|  | MAG (18:3) isomer2 | -1.65 | ↓** | 1.67 | -0.43 | -- | 0.46 |
|  | MAG (18:2) isomer1 | 0.87 | -- | 0.79 | 2.22 | ↑ | 1.15 |
| Vitamins and cofactors | Glutathione reduced form | 0.10 | -- | 0.24 | 1.01 | ↑ | 1.42 |
|  | α-Tocotrienol | -12.20 | ↓ | 1.23 | 0.23 | -- | 0.65 |
|  | Pantothenol | 1.12 | ↑** | 1.65 | 1.26 | ↑* | 1.80 |
|  | -Tocopherol | 1.29 | ↑ | 1.06 | 1.37 | -- | 0.94 |
|  | Menaquinone | 1.19 | ↑ | 1.13 | 0.63 | -- | 0.63 |
|  | Thiamine | 1.97 | ↑ | 1.30 | 0.42 | -- | 0.82 |
| Alkaloids and derivatives | Betaine | 14.27 | ↑* | 1.72 | 0.18 | -- | 0.20 |
|  | 6-hydroxynicotinic acid | 1.20 | ↑ | 1.43 | 0.28 | -- | 0.84 |
|  | Febrifugine | 2.85 | ↑ | 1.23 | 1.24 | -- | 0.86 |
|  | Sophoridine | -1.89 | ↓ | 1.15 | 0.42 | -- | 0.23 |
|  | N-hydroxy tryptamine | -3.46 | ↓ | 1.24 | 1.17 | -- | 0.58 |
|  | N-Acetyl tryptamine | -4.54 | ↓ | 1.18 | 2.05 | -- | 0.74 |
|  | L-Tryptamine | -1.34 | ↓ | 1.09 | -0.35 | -- | 0.52 |
|  | N-Acetyl-5-hydroxytryptamine | -1.28 | ↓ | 1.08 | 0.59 | -- | 0.51 |
|  | 3-Indoleacetonitrile | -1.33 | -- | 0.98 | -1.35 | ↓ | 1.57 |
|  | N-Methyltryptamine | 0.84 | -- | 1.18 | 1.43 | ↑ | 1.54 |
|  | N-Feruloyl serotonin | 0.15 | -- | 0.08 | 2.67 | ↑ | 1.11 |
|  | Theophylline | 0.73 | -- | 0.46 | 1.30 | ↑ | 1.32 |
|  | Caffeine | 0.38 | -- | 0.51 | 1.48 | ↑ | 1.51 |
|  | 1,7-Dimethylxanthine | 0.67 | -- | 0.45 | 1.13 | ↑ | 1.31 |
| Phenylpropanoids and derivatives | Scopoletin | 1.13 | ↑ | 1.44 | 0.22 | -- | 0.34 |
|  | Coniferyl alcohol | 2.24 | ↑ | 1.25 | 0.58 | -- | 0.53 |
|  | *p*-Coumaryl alcohol | 1.29 | ↑* | 1.62 | 0.84 | -- | 0.89 |
|  | 8-Methoxypsoralen | 1.09 | ↑* | 1.65 | 0.41 | -- | 0.61 |
|  | 6-Hydroxymethyl herniarin | -1.02 | ↓ | 1.23 | 0.13 | -- | 0.23 |
|  | Xanthotoxol | -3.95 | ↓ | 1.21 | 1.08 | -- | 0.48 |
|  | Imperatorin | -13.92 | ↓ | 1.23 | 0.23 | -- | 0.12 |
|  | trans-cinnamaldehyde | -3.81 | ↓ | 1.31 | -11.90 | -- | 0.84 |
|  | Syringin | -3.16 | ↓ | 1.42 | 0.09 | -- | 0.07 |
|  | Osthole | -1.81 | ↓* | 1.59 | -0.83 | -- | 0.80 |
|  | O-Feruloyl 2-hydroxylcoumarin | -0.23 | ↓* | 0.66 | -1.49 | ↓ | 1.65 |
|  | trans-4-Hydroxycinnamic acid Methyl Ester | -1.79 | ↓* | 1.61 | 0.78 | -- | 0.82 |
|  | N-p-Coumaroyl hydroxyagmatine | 0.84 | -- | 0.48 | 1.09 | ↑ | 1.50 |
|  | Chlorogenic acid methyl ester | 0.70 | -- | 1.58 | 1.26 | ↑ | 1.55 |
| Flavonoids | 5,7-Dihydroxychromone | 12.77 | ↑ | 1.23 | 0.14 | -- | 0.18 |
|  | Nobiletin | 1.52 | ↑ | 1.17 | 1.40 | -- | 0.96 |
|  | Homoeriodictyol | 1.19 | ↑ | 1.26 | -0.09 | -- | 0.11 |
|  | Tangeretin | 1.45 | ↑ | 1.11 | 1.62 | ↑ | 1.03 |
|  | Kumatakenin | 1.68 | ↑ | 1.17 | 0.74 | -- | 1.63 |
|  | Ayanin | 1.48 | ↑ | 1.27 | 0.51 | -- | 0.69 |
|  | 3-O-Acetyl pinobanksin | 11.00 | ↑** | 1.75 | -0.26 | -- | 0.21 |
|  | Luteolin | -11.08 | ↓ | 1.25 | 1.38 | -- | 0.56 |
|  | Alliin | -9.91 | ↓ | 1.25 | -0.96 | -- | 0.60 |
|  | Pedalitin | -3.82 | ↓ | 1.16 | 1.14 | -- | 0.61 |
|  | 5-O-p-Coumaroylshikimic acid | -2.29 | ↓ | 1.47 | 1.57 | ↑ | 1.45 |
|  | 3-O-p-Coumaroyl shikimic acid | -2.09 | ↓ | 1.58 | 1.04 | ↑ | 1.10 |
|  | Glabridin | -1.89 | ↓* | 1.63 | -0.41 | -- | 0.32 |
|  | Dihydroquercetin | -1.01 | ↓** | 1.69 | -0.24 | -- | 1.27 |
|  | Tricin | -0.74 | -- | 0.64 | 1.39 | ↑ | 1.12 |
|  | Isoliquiritigenin | -0.34 | -- | 0.13 | 1.40 | ↑ | 1.19 |
|  | Catechin | -2.55 | -- | 0.68 | -2.86 | ↓ | 1.16 |
|  | Epigallate catechin gallate | 0.80 | -- | 0.47 | 1.79 | ↑ | 1.02 |
|  | Liquiritigenin | 0.07 | -- | 0.08 | -2.49 | ↓* | 1.74 |
|  | Pinocembrin | 0.21 | -- | 0.27 | -1.21 | ↓ | 1.29 |
|  | Persicogenin | -0.54 | -- | 0.61 | 1.52 | ↑ | 1.28 |
| Glycosides | Narirutin | 2.17 | ↑ | 1.04 | 0.96 | -- | 0.95 |
|  | Tiliroside | 2.25 | ↑ | 1.46 | 0.60 | -- | 0.69 |
|  | Quercetin 3-O-rutinoside | 1.30 | ↑ | 1.39 | 0.37 | -- | 0.22 |
|  | Nicotinic acid-hexoside | -1.70 | ↓ | 1.40 | -0.02 | -- | 0.04 |
|  | Selgin 5-O-hexoside | -2.06 | ↓ | 1.18 | -0.12 | -- | 0.10 |
|  | L-Glutamine O-hexside | -1.97 | ↓ | 1.10 | -0.11 | -- | 0.14 |
|  | 3-O-Feruloyl quinic acid glucoside | -1.10 | ↓ | 1.06 | 0.72 | -- | 1.02 |
|  | Disinapoyl hexoside | -1.95 | ↓ | 1.20 | 0.94 | -- | 1.27 |
|  | Apigenin O-malonylhexoside | -1.35 | ↓ | 1.20 | -1.77 | ↓ | 1.26 |
|  | Tricin 7-O-hexoside | -1.68 | ↓ | 1.11 | 1.03 | ↑ | 1.30 |
|  | Tricin di-O-hexoside | -2.62 | ↓ | 1.15 | -1.00 | -- | 1.22 |
|  | C-hexosyl-apigenin C-pentoside | -3.12 | ↓ | 1.23 | 0.34 | -- | 0.30 |
|  | 6,8-di-C-glucoside Apigenine | -2.48 | ↓ | 1.20 | -0.65 | -- | 1.07 |
|  | Schaftoside | -3.01 | ↓ | 1.22 | 0.17 | -- | 0.15 |
|  | Isoschaftoside | -3.29 | ↓ | 1.23 | 0.06 | -- | 0.05 |
|  | Selgin O-malonyl hexoside | -3.85 | ↓ | 1.16 | -0.03 | -- | 0.02 |
|  | 6-C-hexosyl-luteolin O-hexoside | -1.94 | ↓ | 1.08 | 2.12 | -- | 0.87 |
|  | Chrysoeriol O-malonylhexoside | -2.56 | ↓ | 1.37 | -1.24 | -- | 0.96 |
|  | Vicenin-3 | -2.70 | ↓ | 1.19 | 0.13 | -- | 0.11 |
|  | Isorhamnetin 3-O-glucoside | -3.78 | ↓ | 1.19 | 0.09 | -- | 0.07 |
|  | Isorhamnetin O-hexoside | -2.26 | ↓ | 1.29 | -0.28 | -- | 0.30 |
|  | Isorhamnetin O-acetyl-hexoside | -3.63 | ↓ | 1.20 | -0.17 | -- | 0.15 |
|  | Isorhamnetin 5-O-hexoside | -1.90 | ↓ | 1.05 | -0.46 | -- | 0.49 |
|  | Syringetin 5-O-hexoside | -1.47 | ↓ | 1.15 | 1.51 | ↑ | 1.00 |
|  | D(+)-Melezitose O-rhamnoside | -7.72 | -- | 0.80 | 10.21 | ↑ | 1.41 |
|  | Aspartic acid di-O-glucoside | 0.82 | -- | 1.21 | 2.55 | ↑ | 1.34 |
|  | Nicotinate ribonucleoside | 0.45 | -- | 0.99 | 1.14 | ↑ | 1.66 |
|  | O-p-Coumaroyl quinic acid O-rutinoside derivative | -0.01 | -- | 0.00 | 1.31 | ↑ | 1.61 |
|  | Tricin 4'-O-γ-guaiacylglycerol | -0.15 | -- | 0.25 | 1.41 | ↑ | 1.06 |
|  | Luteolin 7-O-glucoside | -3.31 | -- | 0.75 | 1.20 | ↑ | 1.43 |
|  | Apigenin O-hexosyl-O-pentoside | -0.49 | -- | 1.69 | -1.17 | ↓* | 1.69 |
|  | Kaempferol 3-O-galactoside | -3.42 | -- | 0.73 | 2.34 | ↑* | 1.65 |
|  | Apigenin 7-O-glucoside | -2.07 | -- | 0.69 | 1.33 | ↑ | 1.32 |
|  | Apigenin 5-O-glucoside | -1.85 | -- | 0.58 | 2.26 | ↑ | 1.57 |
|  | C-hexosyl-chrysoeriol O-hexoside | -0.28 | -- | 0.13 | 15.51 | ↑ | 1.12 |
|  | Myricetin 3-O-rhamnoside | 0.37 | -- | 0.33 | 3.33 | ↑ | 1.02 |
|  | Spinosin | 0.42 | -- | 0.46 | -11.19 | ↓** | 1.94 |
|  | Kaempferol 3-O-glucoside | -3.24 | -- | 0.74 | 2.80 | ↑* | 1.77 |
|  | Isoquercitroside | -1.73 | -- | 0.57 | -2.07 | ↓ | 1.09 |
|  | Anthranilate O-hexosyl-O-hexoside | 0.68 | -- | 0.52 | 2.06 | ↑ | 1.26 |
| Peptides | O-Phosphorylethanolamine | -1.03 | ↓ | 1.47 | -0.30 | -- | 0.78 |
|  | Diethanolamine | -1.07 | ↓ | 1.15 | -0.44 | -- | 1.54 |
|  | N'-p-Coumaroyl putrescine | 0.91 | -- | 1.40 | 1.73 | ↑ | 1.49 |
|  | N-p-Coumaroyl putrescine | 0.75 | -- | 1.27 | 1.58 | ↑ | 1.47 |
|  | N-Feruloyl putrescine | 0.56 | -- | 1.09 | 1.36 | ↑* | 1.76 |
|  | 6-Gingerol | -1.09 | ↓ | 1.09 | -2.60 | ↓* | 1.77 |
|  | Phytol | -1.51 | ↓ | 1.30 | -0.54 | -- | 0.88 |
|  | Gallic acid | 0.79 | -- | 0.38 | -13.94 | ↓** | 1.94 |
|  | Ginkgolide A | 0.33 | -- | 1.07 | 1.11 | ↑ | 1.38 |
|  | Chlorpyrifos | 0.00 | -- | 0.00 | -1.06 | ↓ | 1.07 |
|  | 4-Hydroxy benzaldehyde | 1.04 | ↑ | 1.34 | 0.03 | -- | 0.03 |

**Supplemental FIGURE 1** The Main procedures for stepwise MRM (Multiple Reaction Monitoring)-based widely targeted metabolic profiling

Peak alignment and integration

MS/MS spectural tags

(Metware Database)

Peak annotation

(Annotated Metware Database)


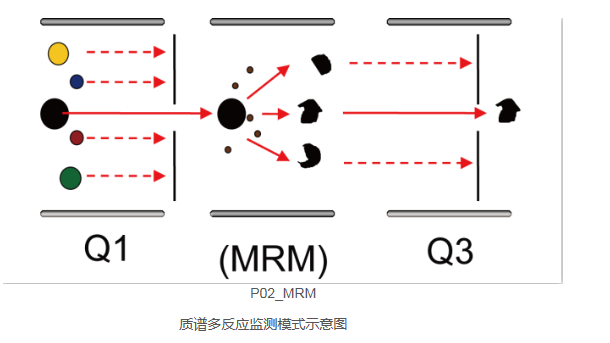


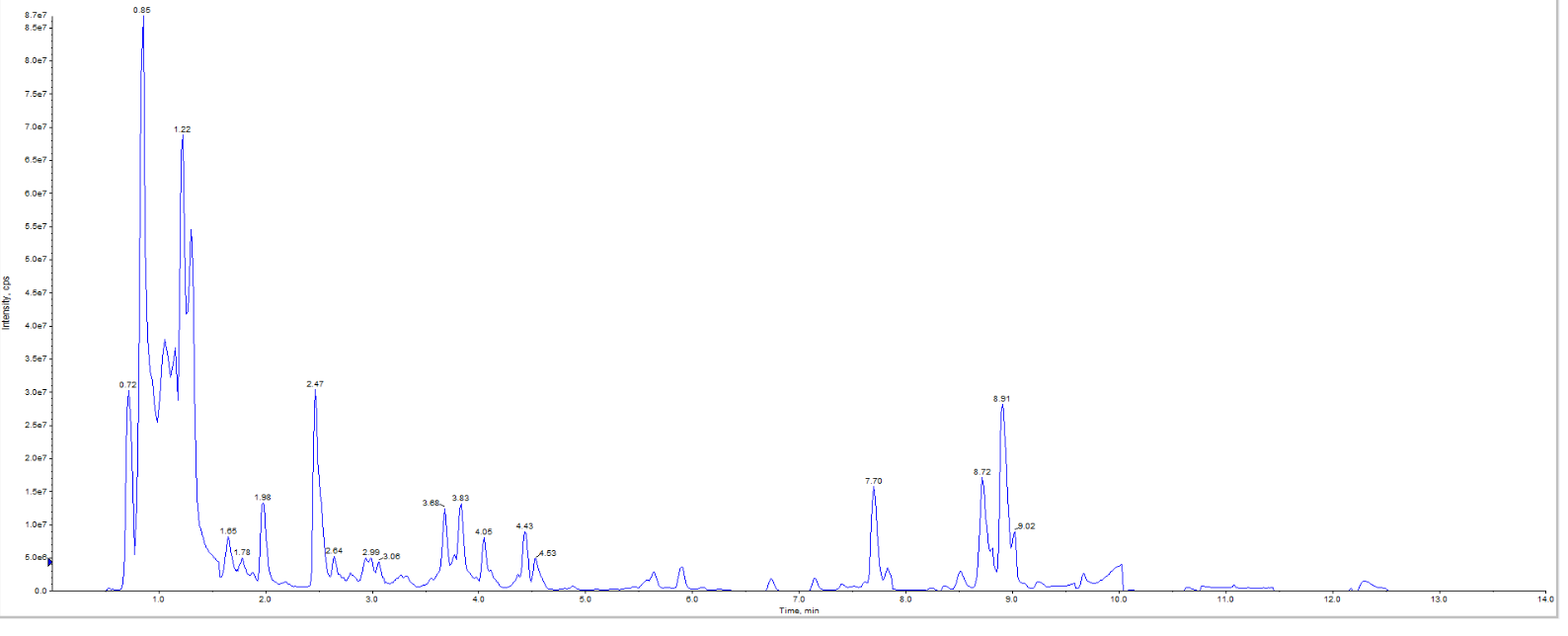


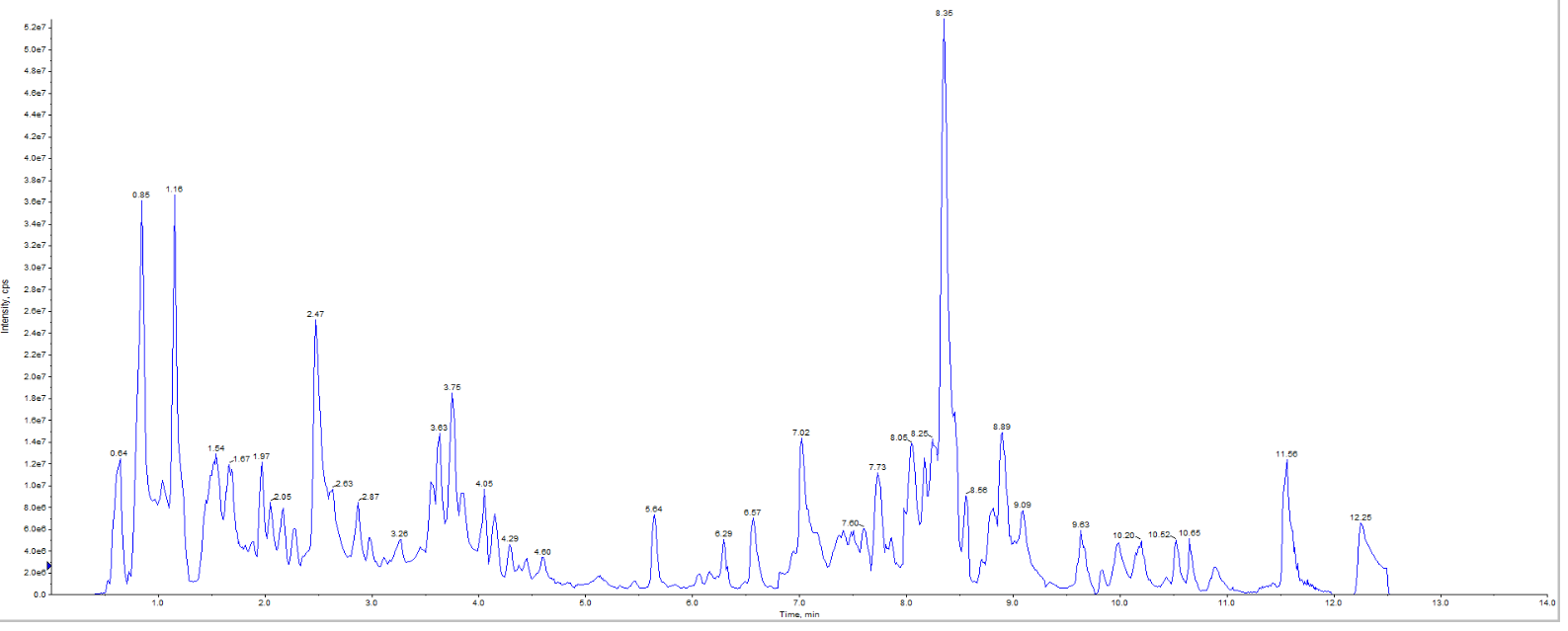


**MS_TIC-N**

**MS_TIC-P**


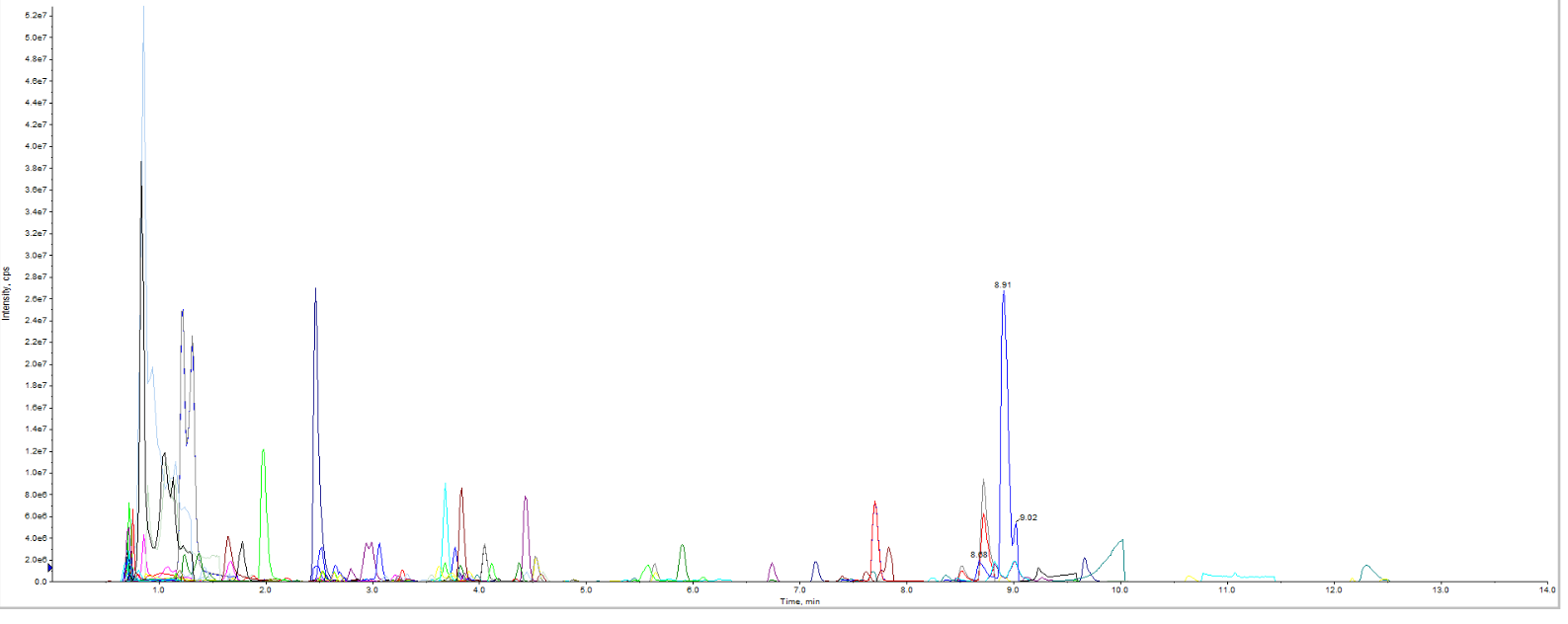


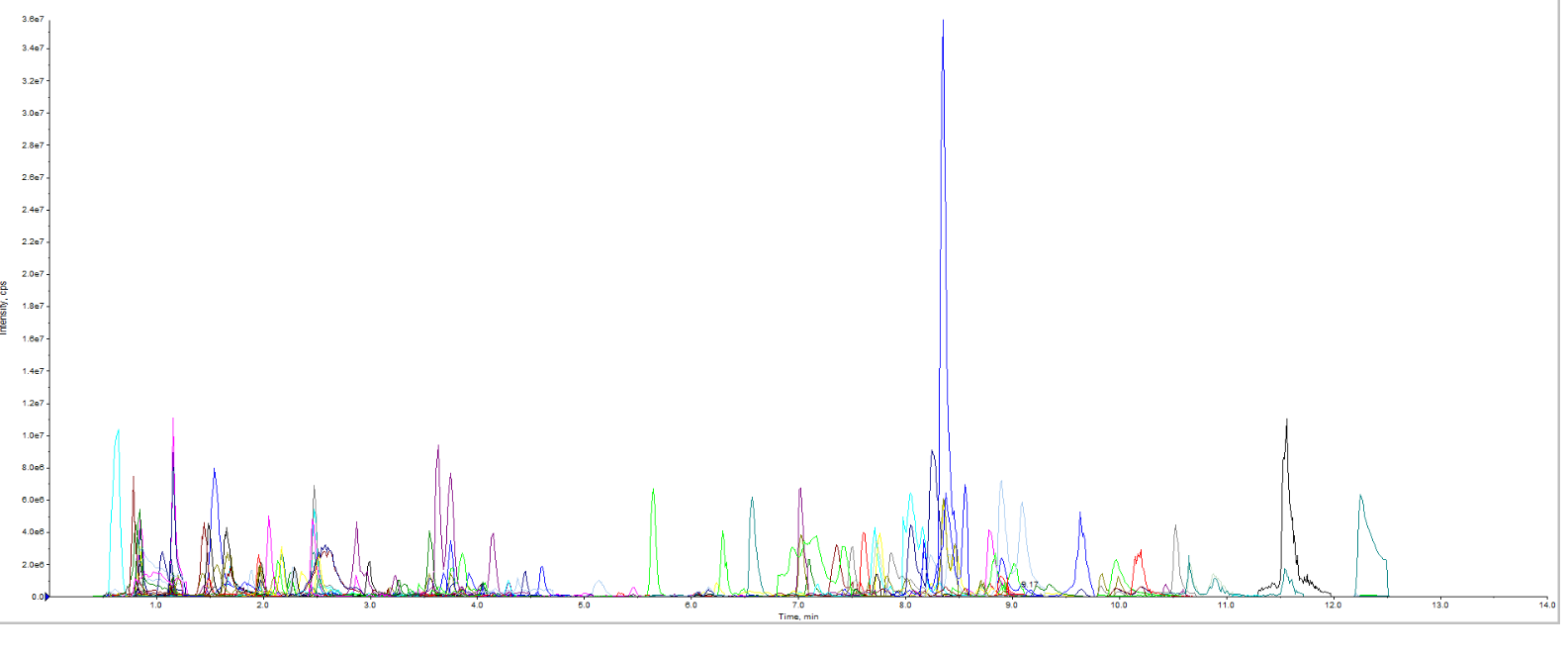


**MRM_XIC-N**

**MRM_XIC-P**

**Total ion chromatography (TIC)**

**MRM-detection of multimodal maps (XIC)**

**Precursor ion screening**

Standard compound Literature search database search

**MRM scan for quantification**

(Annotated Metware Database)

**Supplemental FIGURE 2** Chromatograms of major differential metabolites in roots of Baiyan7 and Yizhangyan4 induced by saline-alkali stress


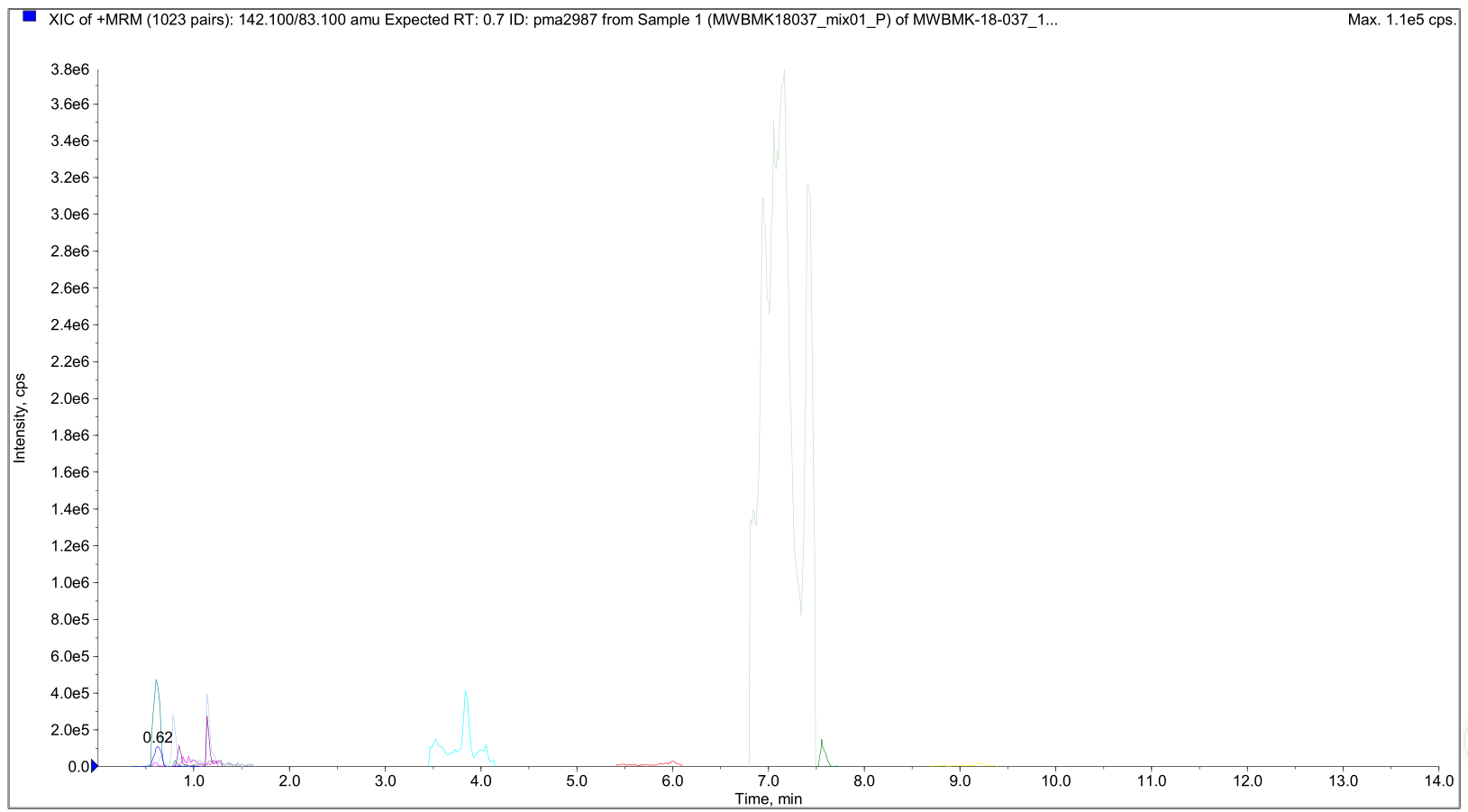

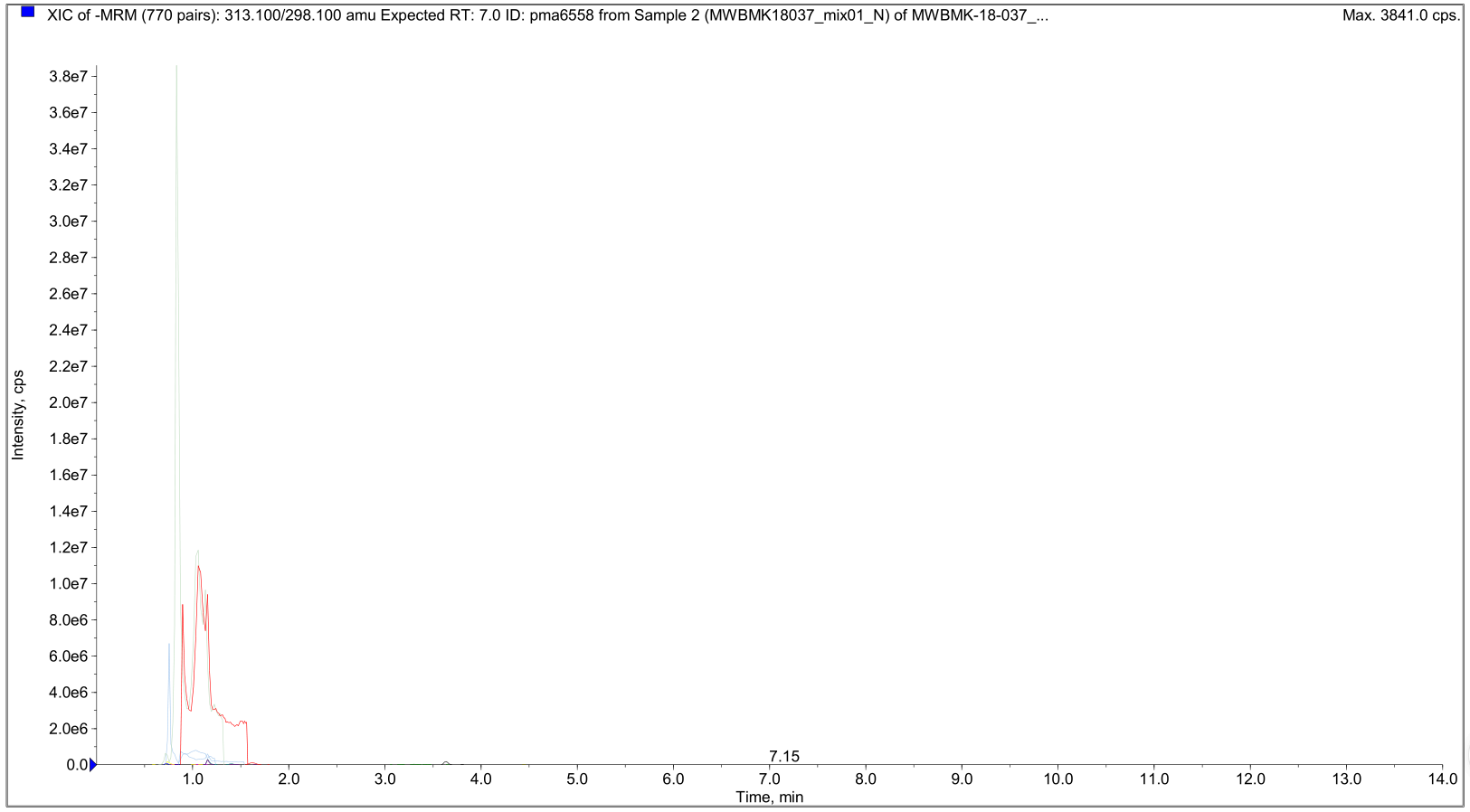

Supplement: Supplementary file 1 [file Data_Sheet_1.docx]
